# Study on the Intelligent Recognition Algorithm for Open-Pit Mine Slope Fissures: Crack-YOLO with Texture and Semantic Enhancement

**DOI:** 10.3390/s26165028

**Published:** 2026-08-07

**Authors:** Hongze Zhao, Hong Wei, Wei Liu, Haiyu Jia, Changbin He

**Affiliations:** 1 School of Energy and Mining Engineering, China University of Mining and Technology (Beijing), Beijing 100083, China; 2 China Coal Pingshuo Group Co., Ltd., Shuozhou 036006, China

**Keywords:** open-pit mine, rock slope, fissure identification, deep learning

## Abstract

Rock fissure parameters, such as length, width, and density, are essential for analyzing the progressive instability of open-pit mine slopes. Under the combined effects of engineering disturbance, geological conditions, and environmental factors, slope fissures continuously propagate and evolve. However, large variations in fissure scale, complex rock-surface textures, blurred boundaries, and weak micro-fissure features increase the difficulty of intelligent fissure segmentation, identification, and parameter extraction. Consequently, many mining enterprises still rely on manual interpretation, which is time-consuming and susceptible to subjective errors. To address these challenges, this study develops Crack-YOLO, a task-oriented fissure detection and instance-segmentation model based on YOLOv8-Seg. A total of 500 original UAV images were collected from multiple open-pit mines and processed to construct a dataset containing 3600 fissure image patches, including 3240 images for training and 360 images for testing. In Crack-YOLO, selected C2f modules are replaced with contextual semantic enhancement modules (CoT Blocks), and a texture information enhancement module (SM Block) is incorporated to strengthen contextual semantic representation and fine-grained texture-feature extraction. The model achieved segmentation precision, recall, mAP50, and mAP50:95 values of 0.896, 0.787, 0.854, and 0.392, respectively. For object detection, the corresponding values were 0.968, 0.862, 0.959, and 0.773, respectively. The segmentation results were further processed using K3M skeleton extraction and physical-scale calibration to quantitatively extract geometric parameters, including fissure length, equivalent average width, and azimuth. Validation using an image containing seven representative fissures yielded mean absolute errors of 0.016 m, 0.010 m, and 0.90° for fissure length, equivalent average width, and azimuth, respectively, indicating the feasibility of the proposed parameter-quantification workflow. In an application test conducted in a typical open-pit mine scene, the proposed workflow identified 196 fissures within approximately 22 s and quantitatively analyzed their geometric parameters and distribution characteristics. The results indicate that the proposed method has potential for fissure identification and geometric-parameter quantification in open-pit mine slopes and may provide quantitative data support for slope-fissure monitoring and stability analysis.

## 1. Introduction

In recent years, rapid advances in machine learning have enabled its extensive application in engineering fields, including geotechnical engineering [1,2,3,4,5] and image recognition [6]. Within this trend toward intelligent engineering, the rapid and accurate characterization of rock structures has become both an important technical requirement and a substantial challenge. Machine-learning methods, particularly deep-learning techniques, have provided new solutions for the automated identification of rock joints and fissures. Through end-to-end feature learning, convolutional neural networks (CNNs) can integrate low-level visual features with high-level semantic information and have demonstrated strong feature-representation capabilities in complex image-recognition tasks [7].

Open-pit mine slope stability is influenced by geological conditions, mining activities, and excavation-induced disturbances [8]. Disturbances associated with the excavation of high and ultra-high slopes may trigger the release of accumulated energy and contribute to progressive deformation or catastrophic slope failure. During this process, existing fissures may propagate and coalesce with adjacent discontinuities, thereby promoting the formation of potential failure surfaces [9]. Accurate fissure identification is therefore important because rock fissures strongly influence the mechanical and hydraulic behavior of rock masses [10].

Rock fissure detection has evolved from traditional image-processing methods to deep-learning-based detection and segmentation. Early approaches mainly relied on threshold segmentation, edge detection, and region growing. Threshold segmentation is computationally efficient and is suitable for images with clear grayscale differences between fissures and the background [11,12]. Edge-based methods can accurately localize fissure boundaries but are sensitive to image noise and heterogeneous surface textures [13,14]. Region-growing methods can preserve regional continuity; however, their performance depends strongly on seed-point selection and similarity thresholds [15]. These limitations become more pronounced in open-pit mine images characterized by uneven illumination, complex rock-surface textures, dust and vegetation occlusion, and substantial variations in fissure scale.

With the development of deep learning, fissure detection has gradually shifted from handcrafted feature extraction to end-to-end feature learning. CNN-based models can automatically extract hierarchical image features, whereas Transformer-based architectures provide stronger long-range contextual modeling. Recent YOLO-based fissure detection methods have mainly been improved through global semantic modeling, multi-scale feature fusion, texture enhancement, and edge-aware feature extraction. For example, Transformer-based neck structures have been introduced to strengthen the global semantic representation of large or through-going fissures [16]. Sobel–Gabor branches and channel-attention mechanisms have been adopted to enhance fissure-edge and texture features [17]. Texture-guided modules, deformable feature extraction, and spatial-context constraints have also been employed to improve the recognition of weak-texture and irregular fissures [18,19,20]. These studies demonstrate the potential of task-oriented feature enhancement for fissure detection under complex backgrounds.

More recent studies have extended fissure detection using two-stage instance-segmentation models, Transformer-based feature encoders, deformable convolutions, lightweight decoders, and multi-scale attention mechanisms [21,22,23,24,25,26,27,28,29,30,31]. Mask R-CNN and related instance-segmentation models can distinguish individual fissure instances but may involve relatively high computational costs. Transformer-based methods provide stronger global contextual representation, whereas deformable convolutions and attention mechanisms improve adaptability to irregular fissure morphologies and multi-scale targets. Lightweight decoders and feature-fusion strategies have also been investigated to improve deployment efficiency. Nevertheless, most existing studies focus on pavement cracks, concrete structures, laboratory rock images, or general industrial defects, and their applicability to complex open-pit mine slope environments remains insufficiently evaluated.

Despite these advances, several challenges remain in open-pit mine slope fissure detection. First, relatively few studies specifically address UAV images acquired from open-pit mine slopes under uneven illumination, dust and vegetation occlusion, and heterogeneous rock-surface textures. Second, micro-fissures and large irregular through-going fissures remain difficult to segment because of their weak texture features, blurred boundaries, and substantial scale variations. Third, most existing studies focus primarily on detection or segmentation accuracy, whereas the conversion of segmentation masks into engineering parameters, such as fissure length, equivalent average width, and azimuth, remains insufficiently investigated. These limitations motivate the development of a task-oriented fissure segmentation and geometric-parameter quantification framework for open-pit mine slopes.

To address these issues, this study develops Crack-YOLO, a task-oriented fissure detection and instance-segmentation model based on YOLOv8-Seg for open-pit mine slopes. The CoT Block and SM Block are introduced to enhance the contextual semantic representation of complex fissures and feature extraction for small and weak-texture fissures, respectively. The instance-segmentation results are further integrated with K3M-based skeleton extraction, eight-neighborhood analysis, and physical-scale calibration to quantify fissure length, equivalent average width, and azimuth. The proposed framework is evaluated through ablation experiments, model comparisons, image-based manual reference measurements, and a field case study.

## 2. Methodology

**Figure 1:**
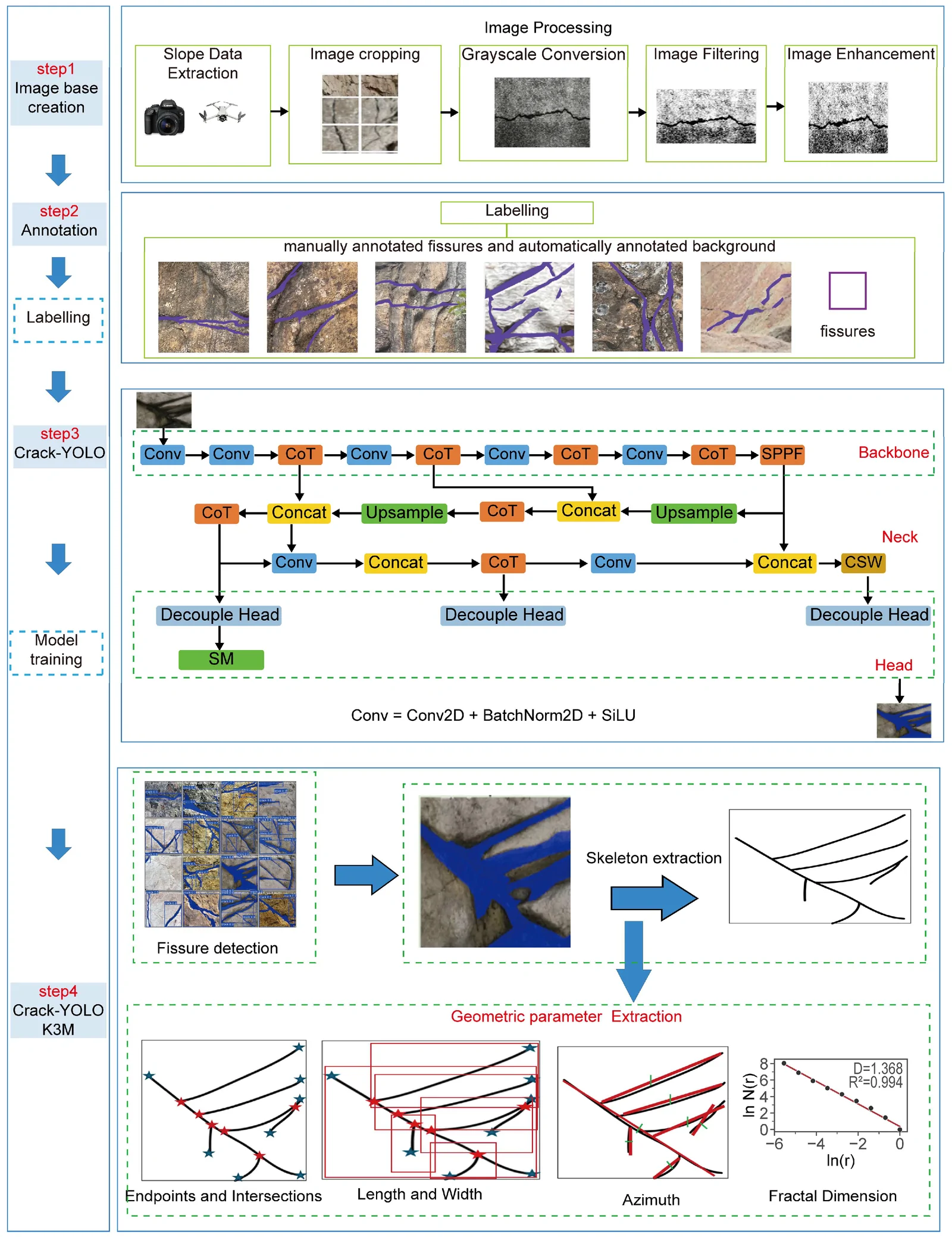
Fissure Identification Process of Crack-YOLO. The blue arrows indicate the overall processing sequence, while the colored boxes distinguish different processing stages and network modules.

Crack-YOLO was developed by modifying the YOLOv8-Seg instance-segmentation framework. In particular, a Texture Enhancement Module (SM Block) is incorporated after the small-target segmentation head of the original YOLOv8 architecture, and a Contextual Semantic Enhancement Module (CoT Block) replaces the original C2f module. This optimization scheme enhances the algorithm’s ability to extract texture features of micro-fissures, thereby greatly improving the modeling performance for complex fissure morphologies. As illustrated in Figure 1, the fissure recognition, segmentation and parameter extraction based on Crack-YOLO are implemented through four key steps [28].

Step 1: Source-Image Grouping and Dataset Partitioning

In this study, UAVs were used to acquire 500 original images of open-pit mine slope rock fissures. Before image cropping, the original UAV images were grouped according to their source identifiers and divided into training and test sets at a ratio of 9:1.

Step 2: Image Processing and Annotation

The images in the two subsets were independently cropped and preprocessed, producing a total of 3600 rock fissure image patches. All patches derived from the same original UAV image were retained within the same subset. The resulting image patches were subsequently annotated using the Roboflow platform, with rock mass and fissures defined as the two target categories.

Step 3: Prediction and Training

After the training dataset was imported into the Crack-YOLO framework, the model could distinguish fissures from the surrounding rock background based on feature patterns in the annotated images. During training, the internal parameters of the model were continuously iterated and updated to enhance its recognition capability for fissures of different scales. Upon completion of training, the test set was used exclusively for the final evaluation of model performance. Crack-YOLO first located fissure targets in the images, and then compared the predicted results with ground truth annotations, so as to evaluate the model’s recognition accuracy and overall performance.

Step 4: Identification and Extraction of Fissures

Crack-YOLO can accurately distinguish the features of complex fissures and rock backgrounds to realize precise segmentation of fissures. Combined with two-dimensional image analysis techniques and the K3M sequential iterative algorithm to extract fissure skeletons, this method acquires core geometric parameters including fissure length, width and azimuth. These parameters serve as an important basis for the prevention and control of open-pit mine slope disasters.

### 2.1. Image Acquisition and Preprocessing

Lightweight UAVs feature high flexibility, enabling them to operate in complex mining areas and capture high-precision images efficiently. In this study, we adopted such UAVs to conduct aerial photography of open-pit mine slopes. To lower the computational memory overhead related to non-fissure pictures, the acquired images were effectively segmented. In order to achieve a ground resolution of 5 cm, which precisely defines the geometric architecture of rock fissures, the UAV flying altitude was set at 100 m, guaranteeing large-area slope coverage.

A total of 500 original UAV images of rock fissures on open-pit mine slopes were collected from multiple open-pit mines. These images covered diverse rock-surface textures, fissure morphologies, illumination conditions, and acquisition scenarios. After image cropping and preprocessing, a dataset comprising 3600 fissure image patches was constructed. All patches derived from the same source image were assigned to the same subset to prevent information leakage between the training and test sets.

In order to create grayscale images for the segmented rock fissure images, a weighted average approach was used. After denoising the images with median filtering, the images were preprocessed using histogram equalization and other image improvement methods.

### 2.2. Annotation and Dataset Partitioning

**Figure 2:**
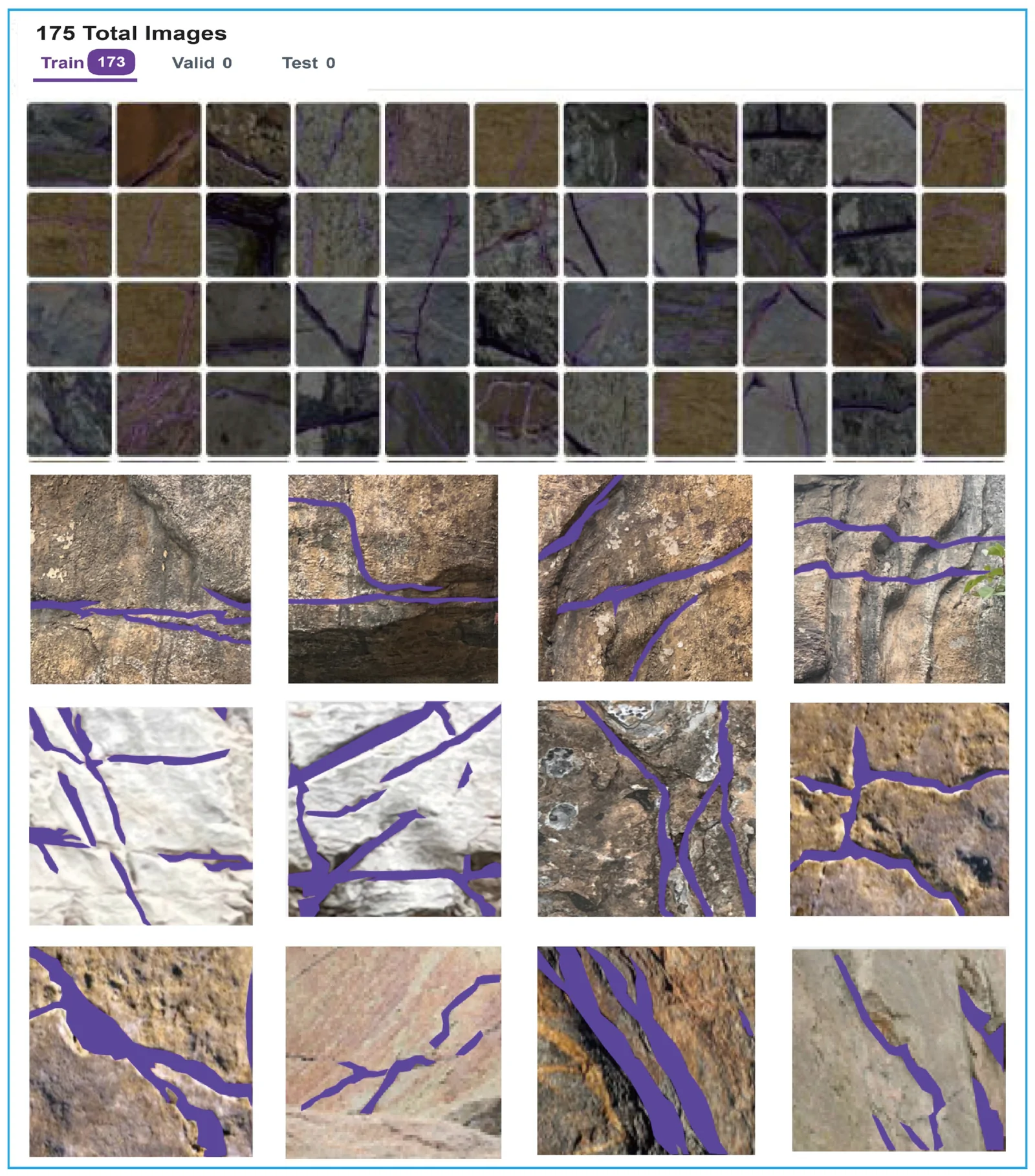
Representative examples of pixel-level annotations for open-pit mine slope fissures. The numerical information displayed in the Roboflow interface is provided only to illustrate the annotation interface and does not represent the final dataset partition used in this study. The image patches were obtained from different source images and therefore are not displayed at a common physical scale. All image patches were resized to 640 × 640 pixels before being input into the network.

The photos of the rock fissures were loaded into the Roboflow platform following the completion of image preprocessing. The strike, length, and width of rock fissures were manually annotated, and the contours of the fissures were marked pixel-by-pixel. Background regions were categorized and annotated using the platform’s integrated smart aided annotation capability, which was based on variations in brightness, texture features, and other attributes. Figure 2 provides examples of annotated images.

Dataset partitioning was performed at the original UAV-image level before image cropping. This strategy was adopted because the number of available original fissure images was limited, and sufficient samples were required for model training while maintaining an independent test set for performance evaluation. The 500 original UAV images were grouped according to their source identifiers and randomly divided into training and testing sets at a ratio of 9:1. This ratio provides a balance between model optimization and independent performance evaluation, which has been commonly used in computer vision-based deep learning studies. All cropped patches generated from the same original UAV image were assigned to the same subset. Therefore, no original image contributed cropped patches to both the training and testing sets, avoiding potential information leakage.

After group-wise partitioning, the images in the two subsets were independently cropped, preprocessed, and annotated. The training set was used for model training and optimization, whereas the test set was reserved exclusively for the final evaluation of model performance. As shown in Table 1, the final dataset contained 3240 cropped image patches in the training set and 360 cropped image patches in the test set, accounting for 90% and 10% of the total dataset, respectively.

### 2.3. Crack-Yolo Fissure Identification Method

Traditional object detection algorithms suffer from inherent technical drawbacks, which can be effectively addressed by the emerging regression-based modeling approach adopted by the You Only Look Once (YOLO) series. YOLO adopts a standalone convolutional neural network to construct an end-to-end detection framework, capable of simultaneously predicting the bounding box coordinates and category probabilities of target objects. The Fully Convolutional Network (FCN) serves as the core structural support for this detection logic. It first partitions input images into evenly distributed grid units, and each unit independently generates a fixed number of bounding boxes, corresponding confidence scores and category probability values. A comprehensive loss function is subsequently adopted to calculate and quantify the errors between predicted results and actual values. As the latest upgraded version of the YOLO series, YOLOv8 was released by Ultralytics in 2023 [29]. Iteratively optimized on the basis of previous frameworks including YOLOv3, YOLOv5 and YOLOv7, this algorithm achieves substantial improvements in both detection precision and computational efficiency. Currently, YOLOv8 stands as one of the most advanced object detection networks. It innovates upon the structural design and optimization strategies of prior models, realizing higher detection accuracy without sacrificing inference speed. This network supports multiple mainstream backbone networks such as ResNet, CSPDarknet and EfficientNet, allowing flexible model selection for different research scenarios and practical demands. To further enhance model robustness and generalization performance, YOLOv8 integrates advanced data augmentation strategies including MixUp and CutMix. It also adopts adaptive training mechanisms to dynamically adjust loss functions and optimize learning rates throughout the training process. Nevertheless, the C2f module of YOLOv8 shows obvious limitations in the recognition task of open-pit mine slope rock fissures. Although this module can complete multi-scale feature fusion, it is constrained by local receptive field characteristics. It fails to effectively capture the long-range feature dependencies of fissure targets, resulting in suboptimal detection performance for complex rock fissures.

As a neighboring region semantic enhancement module, the CoT Block strengthens the network’s semantic perception of fissure areas and improves the extraction accuracy of fissure morphological features, thereby boosting the overall performance of fissure segmentation. Thus, by substituting the CoT Block for the original C2f module in YOLOv8, long-range dependencies are established, the modeling capability for complex fissure shapes is improved, and the network is able to more efficiently aggregate important information in feature maps—demonstrating notable advantages in processing irregular shapes and small fissure targets.

**Figure 3:**
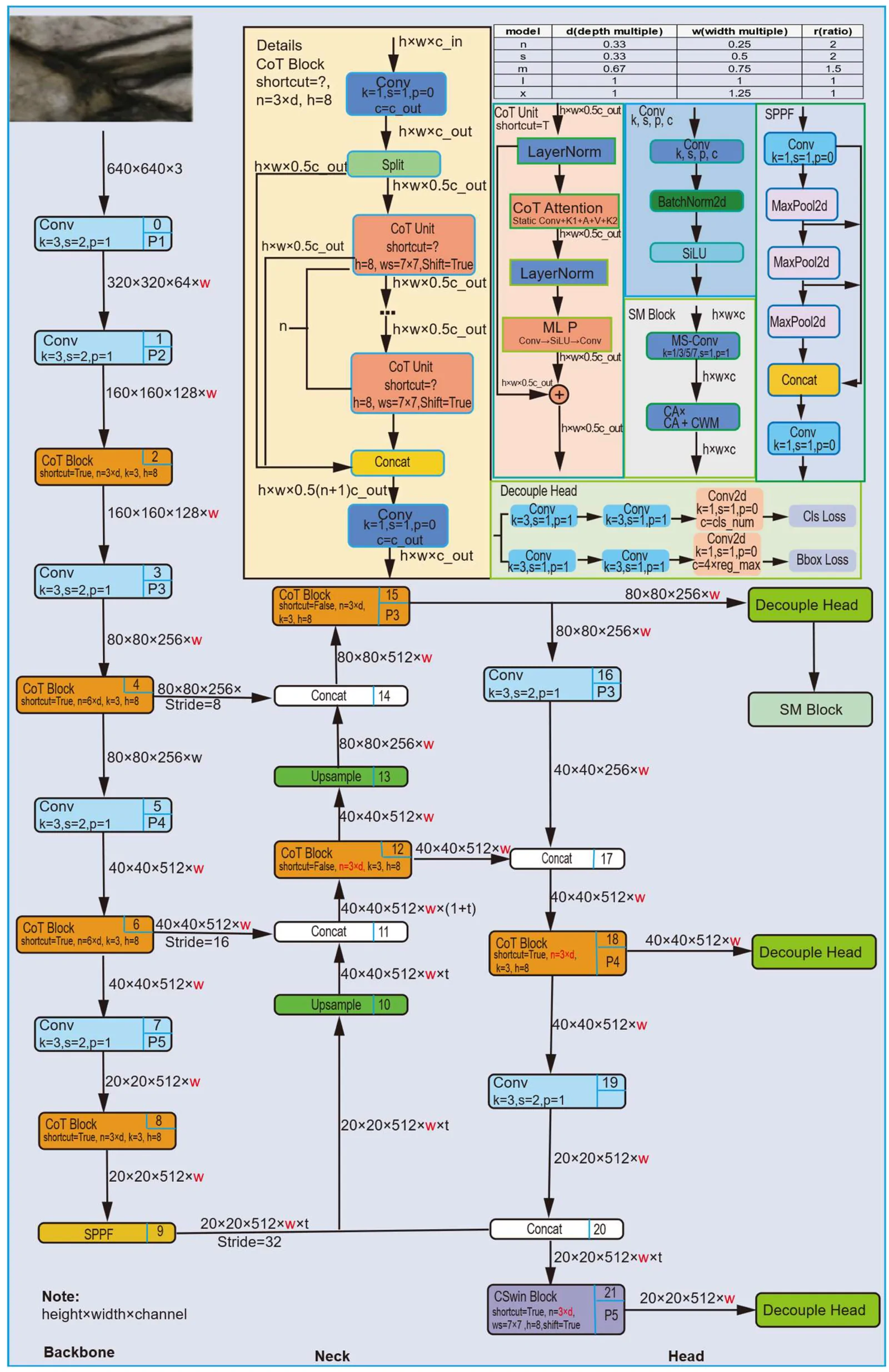
Network Architecture of Crack-YOLO for Rock Fissure Segmentation. Different colors are used to distinguish different network modules and processing operations.

The Crack-YOLO model structure, which is made up of a head, neck, and backbone, is schematically depicted in Figure 3 [30,31]. The CoT Block has replaced the original C2f module of YOLOv8 in the backbone network, which is based on the Darknet 53 architecture. The head module utilizes the YOLOv8 head, while the neck employs a Feature Pyramid Network (FPN) structure. Below, we provide a description of the core modules that constitute the proposed model’s architectural components:

#### 2.3.1. Backbone Network

Crack-YOLO retains the overall backbone architecture of YOLOv8-Seg, while selected C2f modules are replaced with CoT Blocks to enhance neighborhood contextual interactions and improve the semantic representation of cracks with complex morphologies and blurred boundaries. This task-specific modification complements the SM Block, which is designed to strengthen fine-grained, weak-texture, and low-contrast crack features.

The network focuses on collecting shallow edge details of cracks and maintains the native 3 × 3 initial convolutional kernel size of YOLOv8 to prevent redundancy in receptive fields from larger kernels. Additionally, it maintains the traditional four-stage multi-scale feature extraction structure of YOLOv8, which corresponds to various downsampling ratios (8×, 16×, and 32×). Multi-scale convolutional kernels adapted to crack features are used for feature extraction, with a focus on enhancing the representation of fine-grained crack edges and weak texture information. Strided convolutions are used to downsample each feature extraction step, producing multi-scale feature maps that serve as a hierarchical and distinct feature basis for ensuing detection and segmentation tasks.

The CoT Block efficiently captures interactions between local crack properties and global contextual information by modeling semantic correlations within areas, compared with the conventional C2f module. This design greatly improves the feature representation capabilities of the backbone network for crack targets by addressing the C2f module’s inadequacies in extracting semantic characteristics of low-contrast crack targets. It creates a solid feature base for object detection and feature fusion later on.

#### 2.3.2. Neck Module

The neck module of Crack-YOLO is optimized based on the Path Aggregation Feature Pyramid Network (PAFPN) from YOLOv8. This PAFPN architecture adopts a bidirectional feature fusion strategy, which enables interactive transmission and information interaction between high-level semantic features and low-level detail features, and effectively builds an information bridge for multi-scale crack features extracted by the backbone network. While retaining the fine-grained edge information of rock cracks, the module enhances the semantic feature expression of crack targets through an improved path enhancement approach, which achieves balanced fusion between high-level semantic information and low-level detail features. This optimized structure ensures stable and accurate feature input for the subsequent detection head, thus guaranteeing the reliability of final detection results.

#### 2.3.3. Head Module

The essential design of YOLOv8 is carried over into the Crack-YOLO detection head, which is tailored to the features of open-pit mining slope fissures, such as their tiny scale, weak texture, and strong background integration. In the small-target segmentation branch, a Texture Enhancement Module (SM Block) is integrated, forming a dual optimization strategy of “semantics + texture” with the backbone network’s CoT Block. This addresses the issues of detail loss and sparse small-scale fissure features.

The SM Block precisely captures fine-grained edges and weak texture details of fissures by utilizing parallel multi-scale convolution kernels with sizes of 1 × 1, 3 × 3, 5 × 5, and 7 × 7 to extract texture features and a channel attention technique to improve spatial localization [32]. The Proto module and mask coefficients of YOLOv8 are closely integrated with the features produced by the SM Block. A high-precision fissure segmentation mask is finally produced by utilizing the CoT Block’s semantic augmentation capabilities, greatly increasing the segmentation accuracy of weak-texture and small-scale fissures.

#### 2.3.4. Contextual Semantic Enhancement Module (CoT Block)

A Transformer module based on neighborhood self-attention—Contextual Semantic Enhancement Module (CoT Block)—is used to replace the C2f structure in order to address the problems of key information loss and inaccurate boundary localization when YOLOv8’s original C2f structure processes targets with complex geometric features (like fissures). As a result, the network can more efficiently combine important data in feature maps, showing notable benefits while processing small fissure targets and irregular forms.

In addition to boosting the network’s semantic comprehension of fissure areas, the CoT Block also increases the accuracy of fissure morphological feature capture, which helps the network perform better on fissure segmentation tasks in a holistic way. While maintaining computational efficiency, this module significantly improves the network’s detection and segmentation performance for complex fissures. By establishing long-range feature dependencies, it integrates local detail features with global semantic information, strengthening the network’s capability to model complex fissure topological structures. Furthermore, its unique neighborhood interaction mechanism effectively elevates the segmentation accuracy of irregular fissure targets.

**Figure 4:**
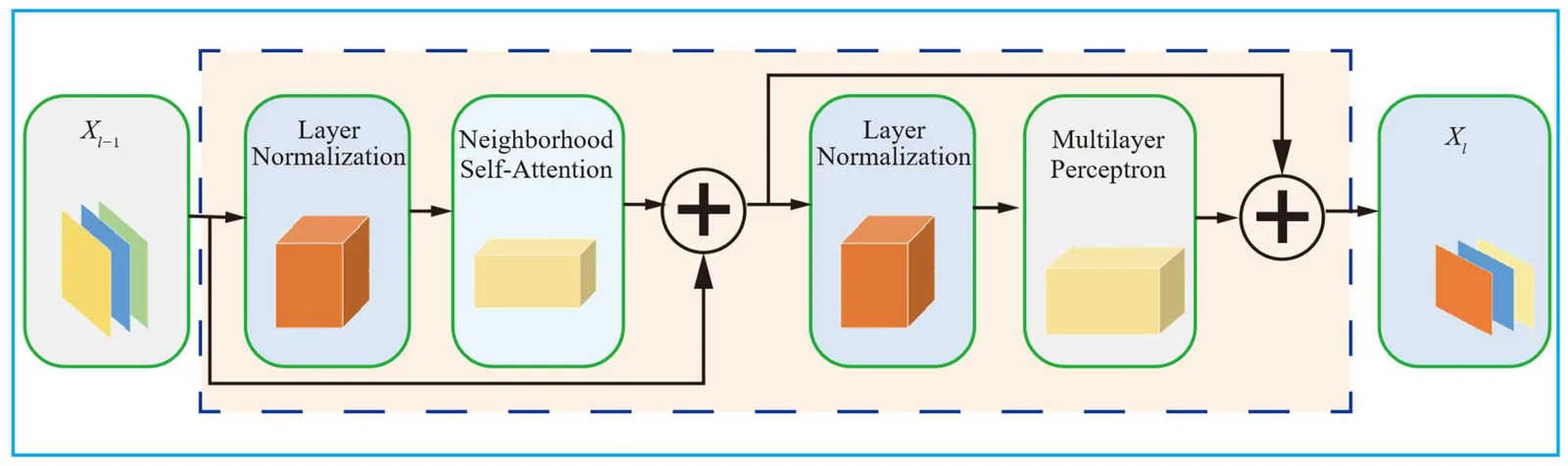
Contextual Semantic Enhancement Module. Different colors distinguish different functional modules and feature representations.

Figure 4 presents the overall structure of the contextual semantic enhancement module. By embedding the neighborhood self-attention mechanism into the Transformer architecture, this module empowers the model with improved capability to extract global semantic information. It features a three-layer structure composed of layer normalization (LN), multilayer perceptron (MLP), and the core neighborhood self-attention mechanism. Residual connections embedded in each LN unit mitigate the vanishing gradient issue during the training of deep networks, thereby ensuring stable model convergence.

Integrated with the GELU activation function and a two-layer MLP structure, the network delivers superior representation performance for complex nonlinear feature correlations. Focusing on refined local feature extraction for fissure regions, the neighborhood self-attention mechanism computes contextual information for all points in the input feature sequence. The calculation process of the Transformer module is defined in Equations (1) and (2) [33].

(1) $${\hat{X}}_{l}=\mathrm{CA}(\mathrm{LN}(X_{l-1}))+X_{l-1}$$

(2) $$X_{l}=\mathrm{MLP}(\mathrm{LN}({\hat{X}}_{l}))+{\hat{X}}_{l}$$

In Equations (1) and (2), $X_{l-1}$ represents the input features, ${\hat{X}}_{l}$ represents the intermediate features, and $X_{l}$ represents the output features of the current layer. The Long-range Dependency Perception Module, which was specifically created, receives the feature map from the architecture. The capacity of the model to identify intricate geometric structures and hazy boundaries is greatly improved by this module, which is essential for capturing global contextual information within the image.

At the same time, the native C2f structures are completely replaced across the network by the Proximity-aware Semantic Enhancement Module, also known as the CoT Block. This replacement highlights the contextual relationships among adjacent regions, allowing for better processing of local detail information.

**Figure 5:**
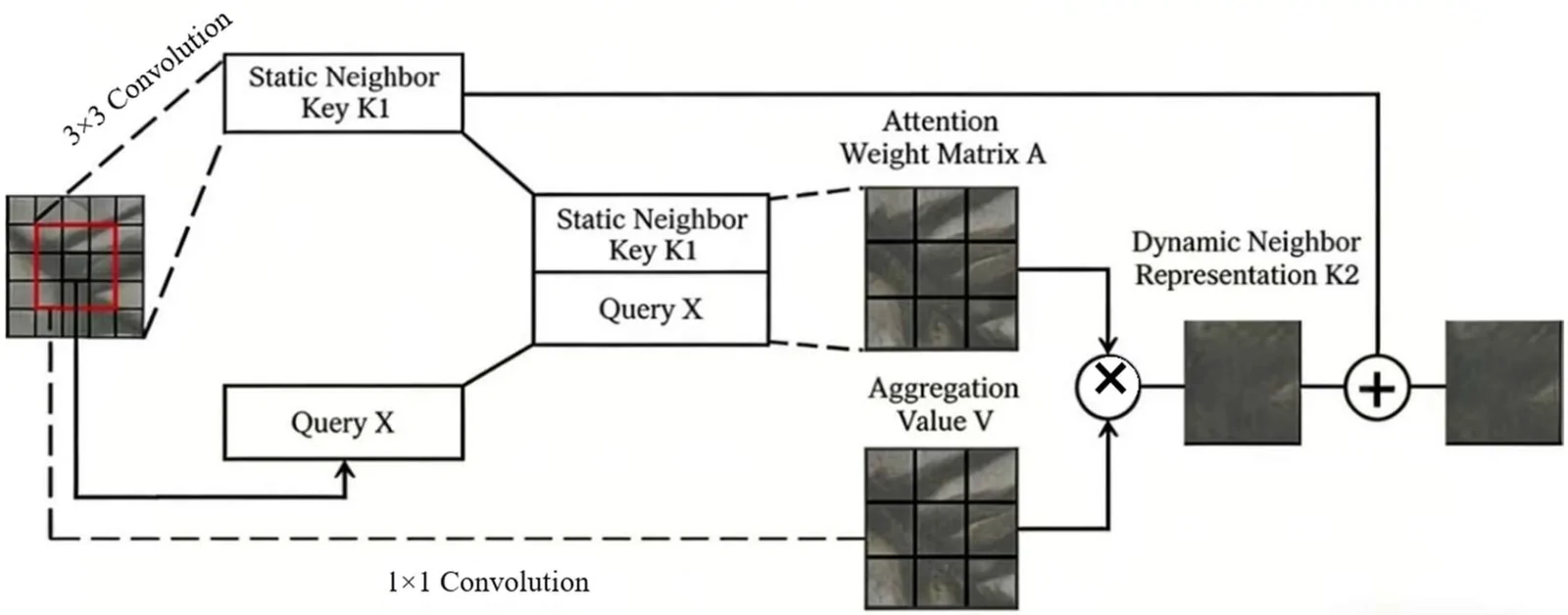
Neighborhood Attention Module.

Serving as the core functional unit of the CoT Block, the proximity-aware self-attention module is dedicated to modeling feature correlations within designated local regions. Its pivotal innovation lies in the integration of self-attention mechanisms and traditional convolution algorithms. The module first applies a 3 × 3 convolution to the surrounding area of each query point, as highlighted in red in Figure 5, to generate static proximity key-value pairs. This process efficiently encodes the spatial structural correlations within local regions and endows the model with fundamental geometric structure perception capability. Subsequently, the query features are concatenated with the generated static key-value pairs, and the resulting fused features are processed through two stacked 1 × 1 convolutions to produce the attention weight matrix. This matrix precisely quantifies the feature interaction strength between query positions and their adjacent regions, thereby establishing dynamic spatial dependencies.

The core advantage of this mechanism stems from the deep integration of self-attention and conventional convolution. A 1 × 1 convolution is used to aggregate the holistic feature information of local regions to obtain integrated local feature values, which are then dynamically weighted by the attention weight matrix to generate context-aware dynamic regional feature representations. The final output features integrate both dynamically adaptive contextual characteristics and static spatial structural information. This architecture can adaptively adjust feature representations according to different query states, significantly enhancing the model’s feature extraction performance in complex scenarios.

Overall, the proposed architecture achieves an optimal balance between dynamic contextual feature updating and static local structure preservation. It combines the adaptive modeling advantages of self-attention with the spatial perception properties of traditional convolution, offering a superior solution for feature extraction tasks in complex rock fissure scenarios. The detailed working mechanism of this module is elaborated in the following section.

The key, query, and value are defined as follows for an input 2D feature map $X\in R^{H\times W\times C}$: the static neighbor key $K_{1}\;$ is generated by **3 × 3** convolution of X, the query Q is obtained by **1 × 1** convolution of X, and $V=XW_{V}$, where $W_{V}$ is a learnable linear weight matrix. In contrast to the standard self-attention mechanism, which encodes each key using a **1 × 1** convolution, the Neighborhood Attention Module generates static neighboring region keys for neighborhood processing by first performing a **k × k** grid convolution on all surrounding keys inside the local neighborhood.

This module uses a feature-learning approach to effectively extract local and regional information. The fundamental input for further feature processing is the static neighboring region keys produced by the **3 × 3** convolution operation, which are essentially a mathematical description of local spatial structures. The module creates the attention matrix during the feature fusion stage by concatenating the static neighbor key $K_{1}\;$ with the query features Q along the channel dimension. Using a specific convolutional processing flow, a **1 × 1** convolution with ReLU activation (Conv1 × 1-ReLU) is utilized for nonlinear transformation initially, and then a **1 × 1** convolution without activation (Conv**1 × 1**-linear) to finish feature mapping. In addition to capturing intricate feature interactions, our approach guarantees that the attention matrix will retain the required linear feature expression capability.

(3) $$A=\mathrm{Softmax}(\mathrm{Concat}(K_{1}\;,Q)W_{\theta }W_{\delta }\;)\;$$

(4) $$K_{2}=V\text{×}A$$

where $K_{2}$ is the dynamic neighborhood representation of the input feature. The final output of the Neighborhood Attention Module is obtained by residual fusion of $K_{2}$ with the original input feature X.

#### 2.3.5. Texture Information Enhancement Module

In image recognition tasks, rock fissures in open-pit slopes present particular difficulties, usually characterized by tiny spatial scales, fuzzy edges, and irregular morphologies. They appear as little pixel patches in high-resolution photos. More importantly, fissure textures frequently exhibit high levels of background fusion, which causes conventional detection and segmentation techniques to be inaccurate. These characteristics need the establishment of efficient detail capture methods and the possession of high-precision feature extraction capabilities in fissure detection algorithms.

Through a specially created small object segmentation head in its multi-branch decoder structure, the YOLOv8-Seg algorithm improves recognition performance for minute cracks. However, enhancing the network’s perceptual capacity for these weak characteristics has emerged as a primary focus of model tuning, as small objects frequently exhibit sparse pixel distributions in feature maps.

**Figure 6:**
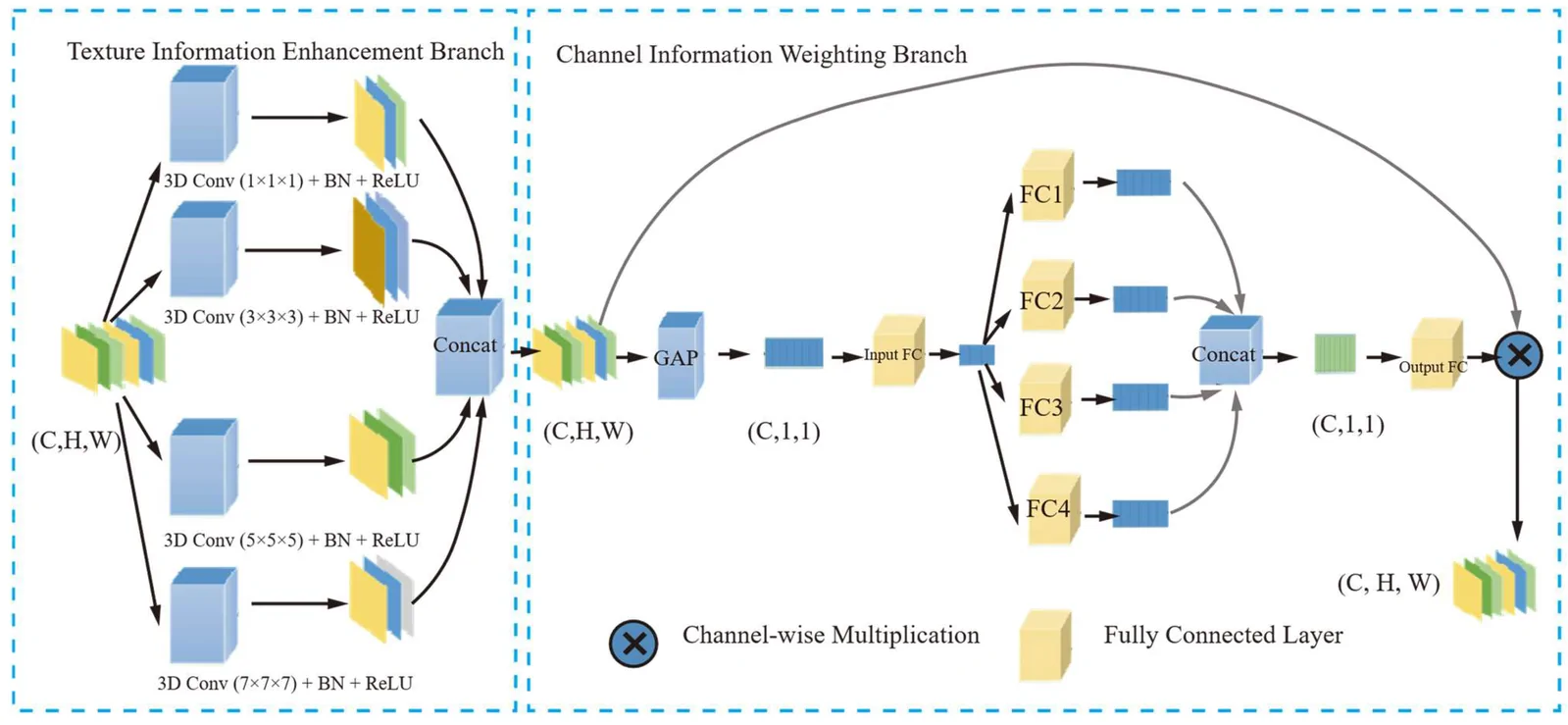
Architecture of the SM Block. Different colors distinguish different functional modules and feature representations.

This work combines multi-scale convolution kernels with a channel attention method to provide fine-grained textural feature extraction in fissure regions, substantially improving the processing capacity for small-scale fissures. Following the tiny object segmentation branch of Crack-YOLO, a Texture Information Enhancement Module (SM Block) is built and introduced, as illustrated in Figure 6. The channel weighting technique improves spatial localization, and the multi-scale convolution structure guarantees the capture of features at various granularities. The network can better detect minute changes in fissure areas by increasing the channel sensitivity of textural characteristics, which solves the issue of insufficient features in small targets.

Four separate branches, each equipped with a convolution kernel of varying spatial dimensions (1 × 1, 3 × 3, 5 × 5, and 7 × 7), concurrently evaluate the incoming feature map, functioning as part of the multi-convolution kernel perception module’s design. Convolution kernels with various receptive fields can fully capture the various fissure texture characteristics, efficiently extract detailed information from numerous scales, and improve the network’s perception of fissure features.

The 1 × 1 convolution kernel is mainly used for extracting the local correlation among feature channels in addition to enhancing feature interaction. Larger-scale convolution spatial filters (5 × 5 and 7 × 7) are deployed to encapsulate wider receptive fields, which improves the architecture’s capacity to comprehend large-scale features. So, this multi-scale feature fusion strategy substantially elevates the resilience and precision of the framework in an effective way when it comes to handle complex and fine-grained targets. Every parallel pathway is assembled using a convolutional operation, Batch Normalization (BN), and a ReLU non-linearity following certain sequence. The input feature map is applied to parallel processing, so that four feature maps with different scale information are finally output.

The resulting quad-scale feature representations are formulated as:

(5) $$U=\mathrm{Concat}(U_{1\times 1},U_{3\times 3},U_{5\times 5},U_{7\times 7})$$

where U_1×1_, U_3×3_, U_5×5_ and U_7×7_ represent the feature maps generated by the 1 × 1, 3 × 3, 5 × 5, and 7 × 7 convolution kernel branches, respectively. Concat means channel-wise concatenation, and $U\in R^{H\times W\times 4C}$ denotes the concatenated feature map (the channel number of each single convolution branch is C). After concatenation, U is fed into the subsequent attention calculation layer.

The second part is the attention calculation layer. The multi-scale feature map $U\in R^{H\times W\times 4C}$ is input into this layer, and processed by a global average pooling (GAP) layer to obtain Spooling $S_{pooling}\in R^{1\times 1\times 4C}$, which is a channel-wise global feature descriptor. Spooling is then input into a fully connected (FC) layer to perform preliminary feature sensing. After that, this process yields four parallel FC branches, and the channel dimension of each branch is reduced to 1/4 of the input channel number to obtain four groups of channel attention weights (where C is the number of input channels of the attention layer). The core objective of the module is to extract multi-dimensional information from the feature map through a parallel branch structure, where each branch can focus on local features at specific scales. Such a design is able to enhance the model’s feature representation capability for rock fissures of different sizes.

The multi-scale feature map $U\in R^{H\times W\times 4C}$ undergoes channel compression through 1 × 1 convolution. As a result, the channel dimensionality is decreased to 25% of its starting magnitude, and $U_{dp}\in R^{H\times W\times C}$ is obtained. The intermediate representation is scaled element-wise by $F_{o}$ across the channel axis to realize dynamic weighting of features. Such design enables the model to strengthen the feature representation of key areas while maintaining computational efficiency. Through the attention-guided feature weighting mechanism, the network enhances the feature response to important areas and suppresses the influence of non-critical areas, thereby improving the discriminability and segmentation performance of the feature map.

The final output feature map $X_{\mathrm{out}}\in R^{H\times W\times C}$ can be expressed as:

(6) $$X_{\mathrm{out}}=F_{o}\times U_{\mathrm{dp}}$$

The Texture Information Enhancement Module (TIEM) adopts a dual-branch architecture, where the first branch performs feature extraction through convolutional filters of varied spatial scales (1 × 1, 3 × 3, 5 × 5, and 7 × 7). Concurrently, the second branch introduces a dense self-selection mechanism into the attention calculation layer, which comprises input–output fully connected layers and four dedicated attention computation layers, so as to select multi-scale features.

For feature processing, the module draws on the channel attention mechanism of SENetV2, and uses four fully connected branches to reduce and concatenate feature dimension. This multi-branch structure allows for feature perception from multi-dimension, preserves the advantage of channel attention weighting while enhancing texture feature representation through multi-scale feature extraction. Ultimately, the module can achieve refined extract and adaptive enhancement of texture information, improving the discriminability of feature representations.

#### 2.3.6. Loss Function

This study introduces Varifocal Loss (VFL) as the core objective function to enhance the segmentation and classification performance of the Crack-YOLO model. VFL focuses on the coordinated optimization of prediction confidence and bounding box localization accuracy. It evaluates localization quality based on the Intersection over Union (IoU) between predicted bounding boxes and ground-truth counterparts. Different from traditional loss functions that rely on fixed sample quantity normalization, VFL leverages IoU values as dynamic weighting factors to optimize model learning. For positive samples with an IoU value greater than zero, the model directly adopts the corresponding IoU value for loss weighting, which avoids the introduction of redundant auxiliary hyperparameters. In contrast, negative samples with a zero IoU value will be suppressed via targeted hyperparameter tuning. This approach effectively weakens the interference of invalid background regions and prevents them from dominating the overall loss. Such an IoU-based adaptive weighting strategy enables the model to prioritize high-quality sample features throughout the training process, thereby achieving more balanced and efficient learning. The mathematical formula corresponding to the Varifocal Loss mechanism is defined as follows [34]:

(7) $$\mathrm{VFL}(p,q)\left\{\begin{array}{l} -q(q\log(p)+(1-q)\log(1-p)\;\;\;\;\;q>0\\ -\alpha p^{\gamma }\log(1-p)\;\;\;\;\;\;\;\;\;\;\;\;\;\;\;\;\;\;\;\;\;\;\;\;\;\;\;\;\;\;\;\;\;q=0 \end{array}\right.$$

In the aforementioned piecewise function, the variable q represents the Intersection over Union (IoU), a prevalent evaluation metric for quantifying the spatial overlap degree between predicted bounding boxes and ground-truth labels. Mathematically, IoU is calculated as the ratio of the intersection area to the union area of two overlapping bounding box regions. Meanwhile, the variable p refers to the predicted classification probability of the model, which is commonly defined as the prediction confidence score.

To further optimize the bounding box regression performance of the Crack-YOLO model, a hybrid loss strategy combining Distribution Focal Loss (DFL) and Complete Intersection over Union (CIOU) Loss is embedded into the model training pipeline.

As a probability-guided focusing loss function, DFL facilitates rapid and stable convergence of model predictions near ground-truth coordinates. It essentially adopts the cross-entropy function to optimize the probability distribution of discrete values adjacent to the labeled coordinate y. This optimization mechanism concentrates the model’s predicted probability density on the true target value, which substantially improves the accuracy of coordinate regression and enables the model to localize target regions with finer spatial granularity. The specific loss formula for bounding box coordinate regression is presented as follows [34]:

(8) $$\mathrm{DFL}(S_{i},S_{i+1})=-((y_{i+1}-y)\log S_{i}+(y-y_{i})\log S_{i+1}$$

In this formulation, y denotes the regression target, while $y_{i}$ and $y_{i+1}$ stand for the adjacent discrete predicted values, and S represents the output probability distribution.

Taking full consideration of geometric characteristics including shape, spatial position and aspect ratio, Complete Intersection over Union (CIOU) Loss serves as a reliable indicator to measure the difference between predicted bounding boxes and ground-truth counterparts. This loss function boasts strong sensitivity to localization accuracy, which allows it to detect tiny positional offsets precisely. Therefore, the overall robustness and localization accuracy for rock fissure targets are greatly improved. The mathematical expression of Complete Intersection over Union (CIOU) Loss is given as follows:

(9) $$L_{\mathrm{CIOU}}=1-\mathrm{IOU}+\frac{\rho^{2}(b,b^{\mathrm{gt}})}{c^{2}}+\alpha v$$

where the width-height consistency penalty term ***v*** and the weight coefficient ***α*** are defined as:

(10) $$v=\frac{4}{\pi^{2}}{\left(\arctan\frac{w^{gt}}{h^{gt}}-\arctan\frac{w}{h}\right)}^{2}$$

(11) $$\alpha =\frac{v}{(1-IOU)+v}$$

In these formulas, w and h represent the width and height of the predicted bounding box, whereas wgt and hgt refer to the corresponding dimensions of the ground-truth box. The term ρ2(b,bgt) is defined as the squared Euclidean distance between the center coordinates of the predicted box b and the ground-truth box bgt. In addition, c stands for the diagonal length of the minimum enclosing rectangle that contains both the predicted and target bounding boxes.

#### 2.3.7. Evaluation Metrics

To fully evaluate the prediction performance of the improved model, five evaluation metrics are adopted: confusion matrix, mAP0.5, mAP0.5:0.95, precision–recall (PR) curve and F1 curve.

As a basic statistical table for evaluating model prediction performance, the confusion matrix arranges ground-truth classes in rows and the predicted categories generated by the model in columns. As presented in Table 2, this matrix serves as the fundamental basis for calculating subsequent quantitative indicators, including mAP@0.5, mAP@0.5:0.95, PR curves and F1 curves.

Precision defines the ratio of genuine positive instances relative to the total set of predictions labeled as positive by the network, which is mathematically formulated as:

(12) $$\mathrm{Precision}=\frac{\mathrm{TP}}{\left(\mathrm{TP}+\mathrm{FP}\right)}$$

Here, TP (True Positives) denotes the number of positive samples that are correctly identified. FP (False Positives) refers to background or irrelevant samples misclassified into the positive category.

Recall evaluates how comprehensively the model can capture all ground-truth positive samples, serving as a key indicator for the completeness of detection. The mathematical formula for Recall is defined as follows:

(13) $$\mathrm{Recall}=\frac{\mathrm{TP}}{\left(\mathrm{TP}+\mathrm{FN}\right)}$$

FN (False Negatives) refers to positive ground-truth samples that are missed and not detected by the model.

Precision and recall present an inherent trade-off in practical detection tasks: enhancing the model’s prediction precision inevitably reduces its recall capability, and the opposite is also true.

Mean Average Precision (mAP), an authoritative benchmark for evaluating detection and segmentation accuracy, is derived from Average Precision (AP). This metric quantifies model performance by comprehensively characterizing the coupling relationship between precision and recall. Specifically, the AP value corresponds to the integral area under the precision-recall (P-R) curve. In the standard recall range of 0 to 1, equidistant sampling is performed to collect peak precision values for interpolation. These discrete sampled values are weighted by the interval of adjacent recall coordinates and accumulated to generate the final AP result.

As a generalized extension of AP, mAP reflects the model’s average detection performance across diverse confidence thresholds. The calculation initiates at a threshold of 0.5 and increases at a fixed step size (0.05 or 0.1), enabling comprehensive and multi-scale quantitative evaluation of the model’s detection capability.

### 2.4. Fissure Skeleton Extraction Using the K3M Sequential Iteration Algorithm and Geometric-Parameter Quantification

**Figure 7:**
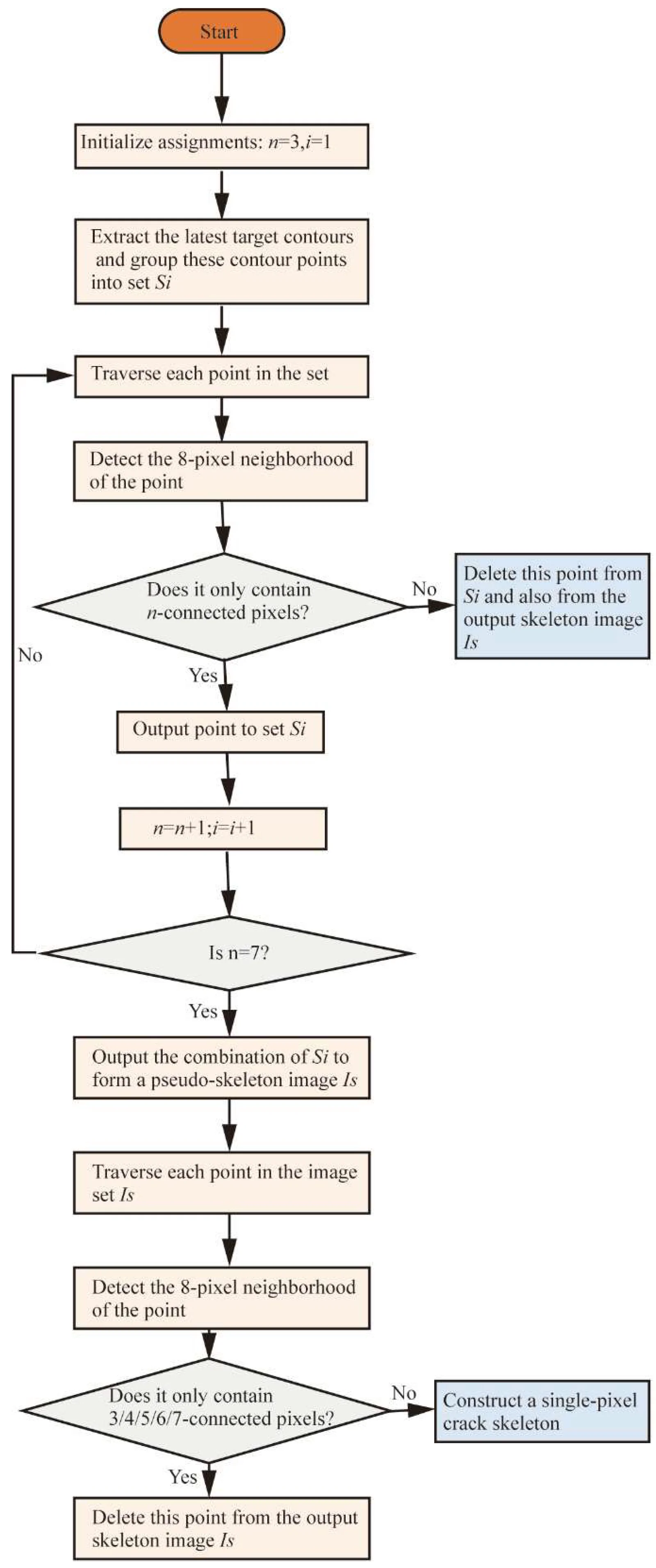
K3M-Based Fissure Skeleton Extraction Workflow.

The pixel-level fissure instance masks generated by Crack-YOLO serve as the direct input to the post-processing quantification module. The K3M sequential iteration algorithm is employed to transform each fissure region with a finite width into a continuous one-pixel-wide skeleton while preserving its principal topological structure and connectivity. The overall workflow is illustrated in Figure 7.

The K3M skeletonization procedure consists of two stages. In the first stage, eligible contour pixels are sequentially removed through iterative contour filtering. When no additional contour pixels can be deleted, the remaining pixels form a pseudo-skeleton of the fissure. In the second stage, the pseudo-skeleton is further refined to eliminate redundant pixels and obtain the final one-pixel-wide fissure skeleton. It should be emphasized that K3M is used only for skeleton extraction. Endpoints, intersection points, and geometric parameters are subsequently obtained by combining the extracted skeleton with the original segmentation mask and additional image-processing methods. The principal parameter-extraction procedures are described below.

(1) Endpoints and intersection points

Endpoints and intersection points are identified from the one-pixel-wide fissure skeleton using eight-neighborhood connectivity analysis. A skeleton pixel is classified as an endpoint when only one neighboring skeleton pixel exists within its eight-neighborhood. When three or more neighboring skeleton pixels are present, the pixel is regarded as a candidate intersection point. Because several adjacent pixels may be repeatedly identified within the same intersection region, the candidate intersection pixels are clustered, and a representative central pixel is retained as the final intersection point. This procedure provides the number and spatial locations of endpoints and intersection points for subsequent analyses of fissure branches and connectivity.

(2) Fissure length and equivalent average width

After the endpoints and intersection points are identified, the intersection points are used as segmentation nodes to divide a complex fissure skeleton into independent branches, thereby avoiding repeated measurements within intersecting regions. The length of a fissure is calculated by accumulating the Euclidean distances between adjacent skeleton pixels:

(14) $$L_{fissure}\;=\sum_{i,j\in C_{1}}l_{ij}$$

where *L*_fissure_ is the total skeleton length of the fissure, *C*_1_ is the set of adjacent skeleton-pixel pairs, and *l_ij_* is the Euclidean distance between adjacent pixels *i* and *j*. For a fissure divided into several branches by intersection points, its total length is obtained by summing the lengths of the corresponding branches.

The fissure area is determined by accumulating all pixels within the corresponding connected fissure region:

(15) $$S_{fissure\;}=\sum_{m,n\in C}p_{mn}$$

where *S*_fissure_ is the fissure area, C is the set of pixels belonging to the fissure mask, and p_mn is the area of the fissure pixel located at (*m*,*n*). In the pixel coordinate system, the area of a single pixel is taken as one pixel squared; therefore, *S*_fissure_ is equal to the total number of fissure pixels within the connected region.

Because fissure width varies along the propagation direction, the equivalent average width is defined as the ratio of the fissure area to its total skeleton length:

(16) $${\bar{W}}_{fissure}=\frac{S_{fissure}}{\mathrm{Sum}(L_{fissure})}$$

where ${\bar{W}}_{fissure}$ denotes the equivalent average fissure width. The conversion of pixel-based length and width measurements into physical dimensions is described in Section 3.5.

(3) Fissure azimuth

After the fissure skeleton is obtained, a straight line is fitted to the skeleton pixels of each fissure using the least-squares method:

(17) $${\hat{y}}_{i}=a+bx_{i}$$

where $x_{i}$ and ${\hat{y}}_{i}$ are the horizontal and fitted vertical coordinates of the skeleton pixels, respectively, *a* is the intercept, and *b* is the slope of the fitted line.

The fissure azimuth is defined as the clockwise angle between the principal propagation direction of the fissure and geographic north, with a range of 0–180°. When the upward direction of the image is aligned with geographic north, the azimuth is calculated as:

(18) $$\theta =\;\mathrm{mod}\;\left[90^{{}^{\circ}}+\arctan(b),180^{{}^{\circ}}\right]$$

where *θ* is the fissure azimuth. When the image orientation is not aligned with geographic north, the calculated angle is corrected according to the calibrated north direction.

(4) Surface fissure ratio

The surface fissure ratio is used to characterize the proportion of the investigated region occupied by fissures. Based on the fissure masks generated by Crack-YOLO, it is calculated as the ratio of the total fissure area to the area of the region of interest:

(19) $$K_{f}=\frac{S_{fissure}}{S_{\mathrm{ROI}}}\times 100\%=\frac{N_{fissure}}{N_{\mathrm{ROI}}}\times 100\%$$

where $K_{f}$ is the surface fissure ratio; $S_{fissure}$ and $S_{\mathrm{ROI}}$ are the total fissure area and the area of the region of interest, respectively; and $N_{fissure}$ and $N_{\mathrm{ROI}}$ are the corresponding numbers of fissure pixels and total pixels. A larger surface fissure ratio indicates a higher degree of surface fissure development.

(5) Fractal dimension

The box-counting method is used to quantify the spatial complexity of the fissure network. Square boxes with a normalized side length r are used to cover the fissure skeleton, and the number of boxes containing at least one fissure pixel, denoted by N(r), is recorded. The fractal dimension is defined as:

(20) $$D=-\underset{r\to 0}{\lim}\frac{\ln N(r)}{\ln r}$$

In practical calculations, several box sizes are selected, and a linear relationship is fitted between ln N(*r*) and ln *r*:

(21) lnN(r)=klnr+c

where *k* is the slope of the fitted line and *c* is the intercept. The box-counting fractal dimension is therefore obtained as:

(22) D=−k

A larger fractal dimension indicates a more complex spatial distribution and a higher branching degree of the fissure network.

Overall, Crack-YOLO provides the pixel-level fissure masks, K3M extracts the corresponding one-pixel-wide skeletons, and the subsequent image-processing procedures quantify the topological, geometric, spatial-distribution, and complexity characteristics of the fissures. This establishes a continuous technical workflow from fissure recognition and segmentation to geometric-parameter quantification.

## 3. Results and Discussion

For the Crack-YOLO model, both the model training process and the corresponding prediction tasks are carried out on a high-performance computing platform. The hardware system adopts a quad-GPU parallel architecture (four NVIDIA GeForce RTX 3090 graphics cards, each with 24 GB VRAM, totaling 96 GB VRAM). The platform is also equipped with an Intel Xeon Platinum 8280L server processor (2.60 GHz), whose multi-core architecture enables efficient parallel computing for deep learning. The system runs on a Linux environment, which is compatible with the PyTorch 1.10 framework. It uses Anaconda3 for environment management. TensorBoard is integrated to visualize and monitor the training process, so as to enhance the efficiency of debugging and optimization. By doing so, there will be continuous improvement of the algorithms on the identification and segmentation of rock fissures in open-pit slopes.

### 3.1. Crack-YOLO Training Results

**Figure 8:**
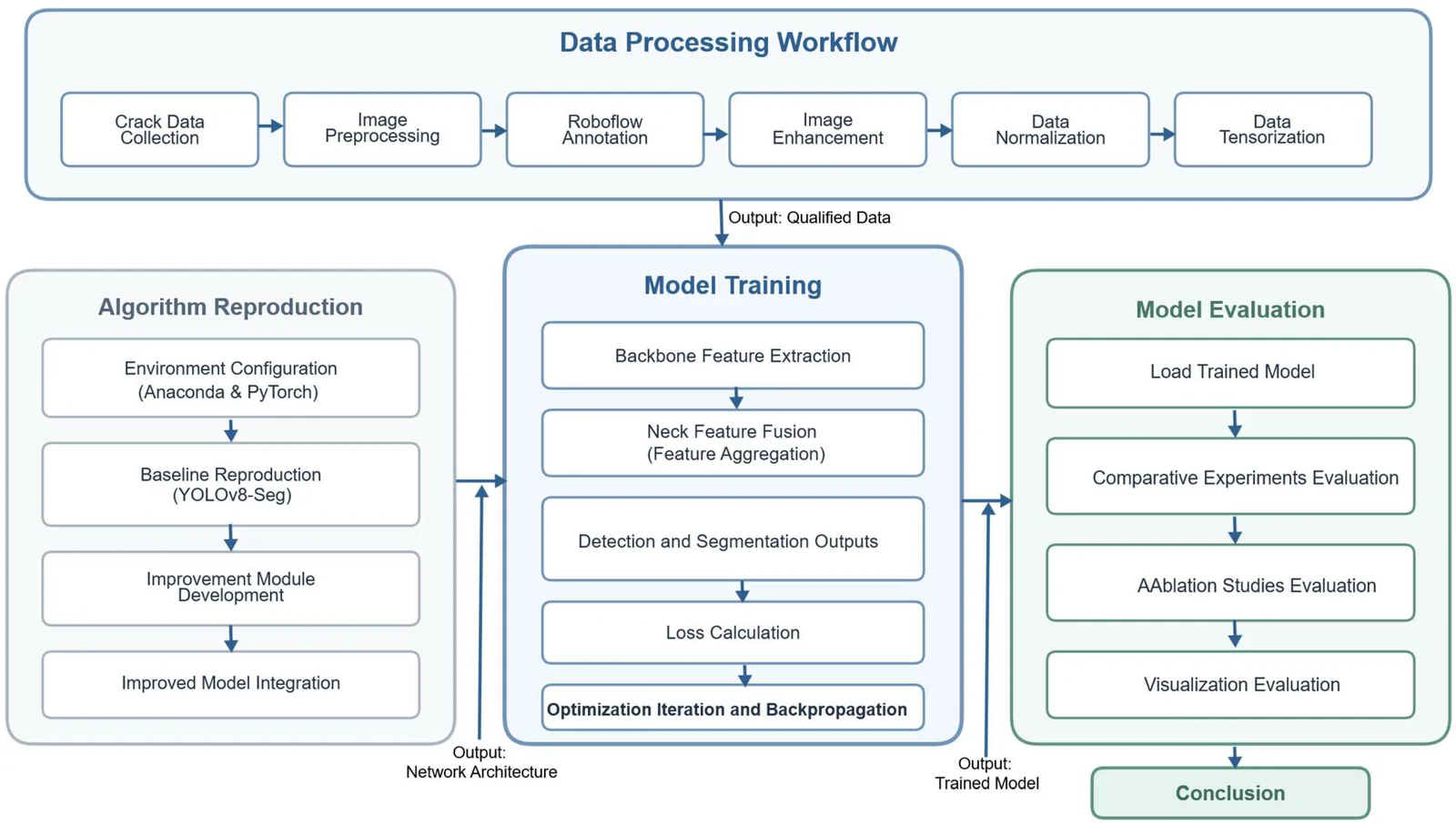
Crack-YOLO Experimental Workflow.

The training pipeline of the proposed Crack-YOLO model consisted of three sequential stages: data processing, model training, and performance evaluation, as visualized in Figure 8. In the data processing stage, dataset acquisition, purification and augmentation are implemented to guarantee high-quality and complete input feature information. The processed datasets are subsequently configured in the YOLOv8 configuration file and standardized through a dedicated preprocessing pipeline, enabling stable and normalized data input throughout the training optimization process. During model training, the YOLOv8-Seg baseline was constructed using the PyTorch framework, and the CoT and SM Blocks were incorporated into the network. All models were trained for 200 epochs with an input size of 640 × 640 pixels and a batch size of 16. The SGD optimizer was adopted with an initial learning rate of 0.01, a final learning-rate factor of 0.01, a momentum of 0.937, a weight decay of 0.0005, and a warm-up period of three epochs. The random seed was set to 0. Data augmentation was applied only to the training set, including Mosaic augmentation (1.0), random horizontal flipping (0.5), translation (0.1), scaling (0.5), and brightness adjustment (0.4). The models were initialized using COCO-pretrained YOLOv8-Seg weights and subsequently fine-tuned end-to-end without layer freezing, while the newly introduced CoT and SM Blocks were randomly initialized. Upon the completion of training convergence, the optimal model weights are preserved. In the final testing stage, quantitative metrics including precision, recall and mAP were evaluated using the test dataset, and the model’s practical inference efficiency and hardware resource utilization were further analyzed.

The Training loss function adopted in this model comprises three core modules: Bounding Box Loss (box_loss) for measuring the spatial offset between predicted bounding boxes and ground-truth coordinates, Segmentation Loss (seg_loss) for evaluating pixel-level segmentation accuracy, and Classification Loss (cls_loss) for quantifying the credibility of object category identification. During the initial training stage, all loss curves drop sharply with iterative parameter optimization. The loss trends gradually level off after around 200 epochs, marking the convergence of the model.

**Figure 9:**
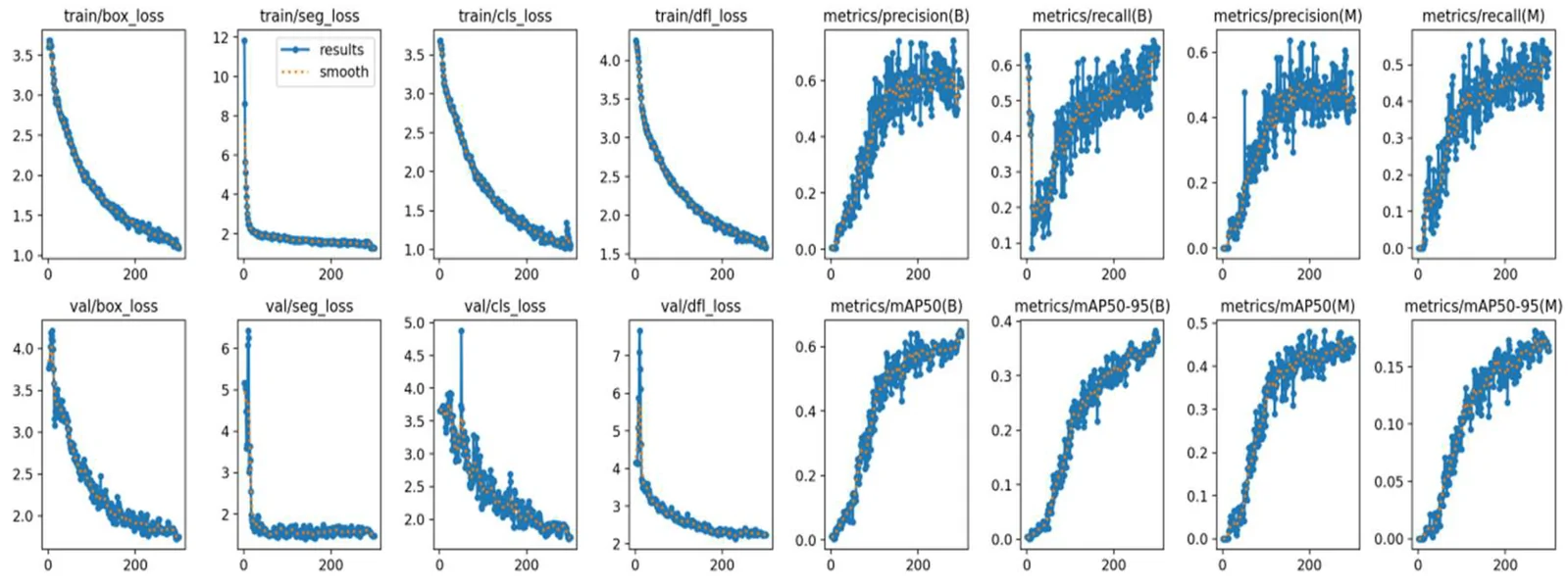
Crack-YOLO Model Training Loss Convergence and Performance Evaluation Metric Curves.

The stabilized loss values at the 200-epoch stage confirm the full maturation of the training procedure, further validating the robust instance segmentation capability of the optimized Crack-YOLO model (Figure 9).

### 3.2. Ablation Study Results and Discussion

The ablation study included four configurations: the baseline YOLOv8-Seg model, the baseline with only the CoT Block, the baseline with only the SM Block, and Crack-YOLO incorporating both modules. This design enables the individual and combined contributions of the two modules to be evaluated. Ablation experiments quantitatively reveal the individual efficacy of the two integrated modules in improving the overall performance of the YOLOv8-Seg baseline. Separate assessments of the Contextual Transformer (CoT) Block and SM Block were conducted to explore their respective optimization effects on key indicators (Precision, Recall, and mAP50), which effectively validates the rationality and superiority of the Crack-YOLO architecture. These empirical findings provide a reliable quantitative foundation for subsequent model refinement and algorithm improvement. Table 3 summarizes the benchmark performance of various advanced detection and segmentation frameworks.

Quantitative data in Table 3 intuitively illustrate the performance variations of the Crack-YOLO model with different module configurations, demonstrating the respective contributions of each improved component to enhancing instance segmentation accuracy. The baseline model without CoT and SM Blocks presents limited detection and segmentation performance. Specifically, its segmentation metrics are Precision = 0.451, Recall = 0.521, mAP50 = 0.450, and mAP50:95 = 0.172, while the corresponding detection metrics are Precision = 0.581, Recall = 0.596, mAP50 = 0.636, and mAP50:95 = 0.382.

The single introduction of the CoT Block yields substantial performance gains. The segmentation indicators are greatly improved to 0.753 (Precision), 0.748 (Recall), 0.740 (mAP50), and 0.322 (mAP50:95). Meanwhile, the detection performance is also significantly optimized, with Precision and Recall both rising to 0.819, mAP50 increasing to 0.891, and mAP50:95 reaching 0.660. This validates that the CoT Block can effectively strengthen the network’s ability to model long-distance spatial dependencies and extract subtle semantic features from complex fissure scenarios.

By solely adding the SM Block, the model achieves further advancement in segmentation precision. The segmentation Precision, mAP50 and mAP50:95 are promoted to 0.833, 0.780 and 0.331, despite a minor decline in Recall (0.670). In the detection task, the model realizes a remarkable Precision improvement up to 0.934, accompanied by increased Recall (0.753), mAP50 (0.907) and mAP50:95 (0.668). The results reveal that the SM Block effectively enhances the model’s texture feature perception capability, which is conducive to capturing small fissure targets that are difficult to identify.

Notably, the combination of CoT and SM Blocks enables the model to reach the best comprehensive performance. In terms of segmentation performance, the model achieves a Precision of 0.896, Recall of 0.787, mAP50 of 0.854, and mAP50:95 of 0.392. For detection performance, the optimal values of Precision (0.968), Recall (0.862), mAP50 (0.959), and mAP50:95 (0.773) are obtained. The two modules produce a favorable synergistic effect, which greatly enhances the network’s multi-scale feature learning capability and refined segmentation performance for complex fissure targets. It can be concluded that the two functional modules complement and promote each other in feature extraction and optimization, thereby comprehensively and effectively improving the instance segmentation and detection performance of the proposed Crack-YOLO model.

### 3.3. Visualization-Based Metric Evaluation Results and Analysis

To conduct a multi-dimensional and comprehensive evaluation of the proposed fissure-segmentation framework, this study employs multiple visual diagnostic approaches, such as confusion matrices, F1-score trajectories, and precision-recall (PR) curves. These visual metrics facilitate systematic and in-depth analysis of the model’s feature discrimination and classification performance.

(1) Confusion Matrix

The confusion matrix establishes a comparative correspondence between ground-truth labels and predicted categories, which intuitively visualizes the model’s classification accuracy. In binary classification scenarios, this evaluation tool is built upon four core statistical components: True Positives (TP), True Negatives (TN), False Positives (FP), and False Negatives (FN). These foundational metrics support the calculation of mainstream evaluation criteria including precision, recall, accuracy, and F1-score, allowing for a refined and comprehensive quantification of the model’s discriminative performance across complex and heterogeneous fissure samples.

**Figure 10:**
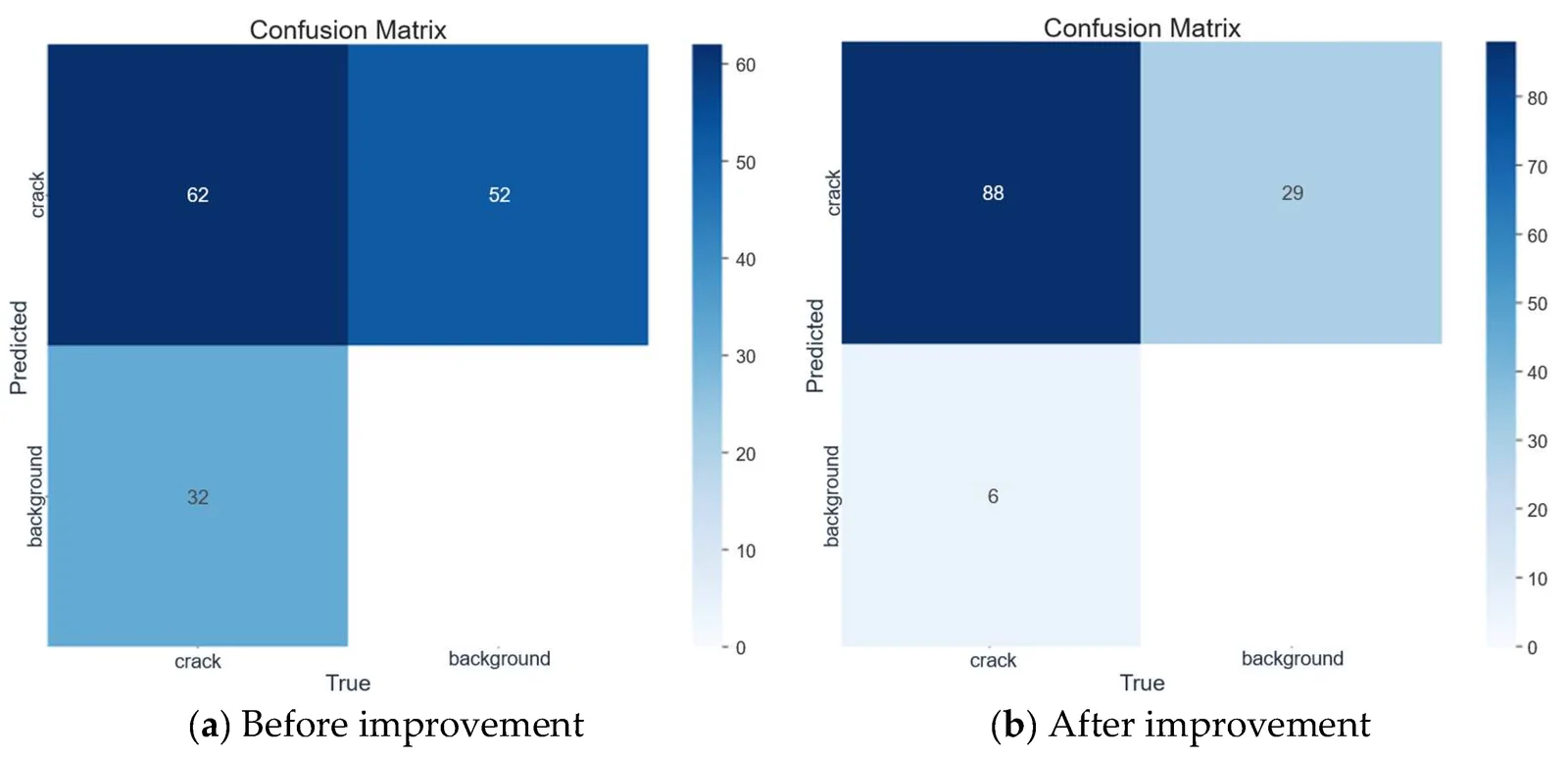
Comparison of Confusion Matrices Before and After Improvement for Crack-YOLO.

Figure 10 presents a comparative confusion matrix analysis of the original and optimized Crack-YOLO models. The quantitative results reveal a prominent increase in true positive (TP) samples, rising from 62 to 88, which verifies the enhanced sensitivity of the optimized model for rock crack detection. Furthermore, the refined Crack-YOLO achieves superior recognition capability for various crack features and substantially decreases false negative (FN) predictions. Accordingly, the overall detection precision and diagnostic reliability of the proposed framework are effectively improved.

(2) F1 Curve

**Figure 11:**
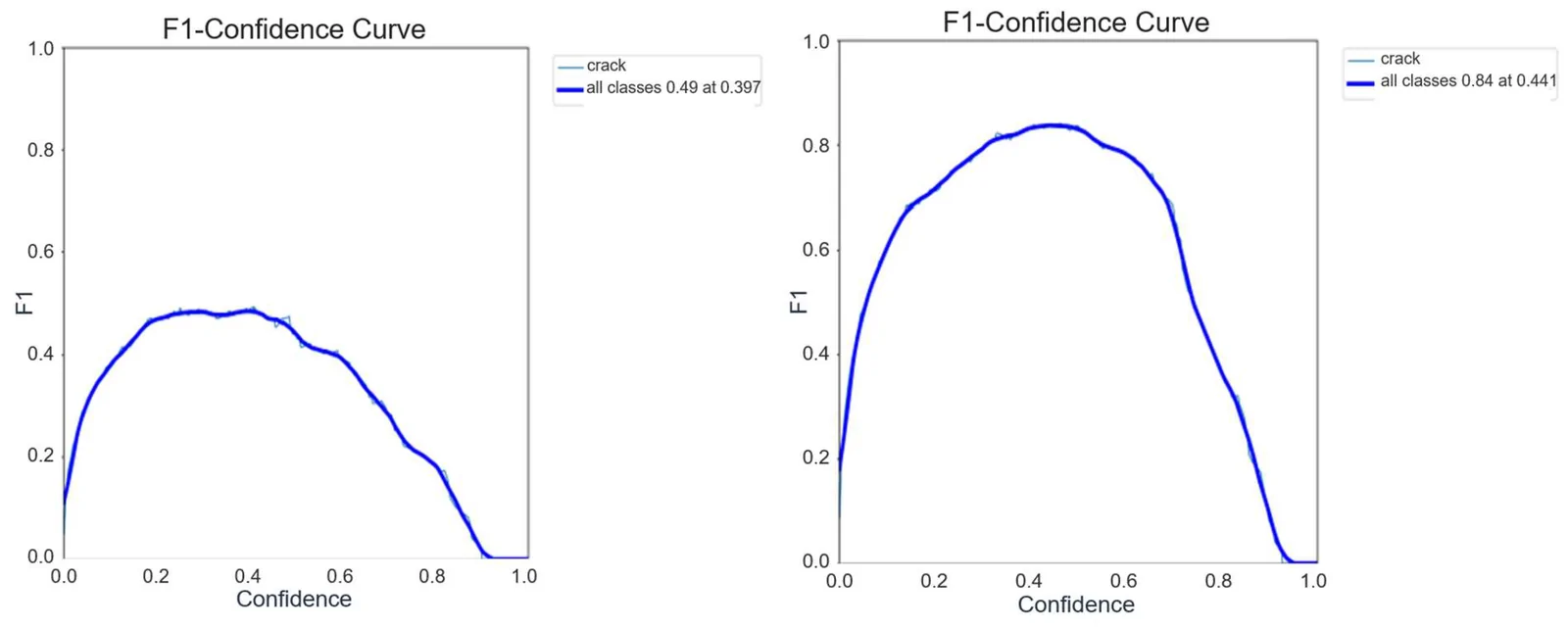
Comparison of F1 Curves Before and After Improvement for Crack-YOLO.

As the harmonic mean of precision and recall, the F1 score acts as a robust integrated indicator to comprehensively evaluate the model’s classification and detection performance. Normalized within the range of [0, 1], a higher F1 score represents a better trade-off between precision and recall and more reliable prediction results. Figure 11 illustrates the F1 curve comparison between the original baseline and the optimized Crack-YOLO model.

The unoptimized baseline model yields a low F1 score of 0.49 at a confidence threshold of 0.397, exhibiting a severe imbalance between precision and recall. In contrast, the proposed Crack-YOLO achieves a significantly improved F1 score of 0.84 at a confidence threshold of 0.441. This substantial performance improvement verifies the efficacy of the designed Adjacent Area Semantic Enhancement Module and Texture Information Enhancement Module, further demonstrating the outstanding capability of Crack-YOLO for high-precision rock fissure segmentation.

(3) Precision-Recall (PR) Curve

The Precision–Recall (PR) curve is an important metric for evaluating segmentation performance, particularly for datasets with imbalanced foreground and background samples. By illustrating the relationship between precision and recall at different confidence thresholds, the PR curve reflects the trade-off between correctly identified fissure pixels and false-positive predictions. A curve closer to the upper-right corner generally indicates a better balance between precision and recall.

**Figure 12:**
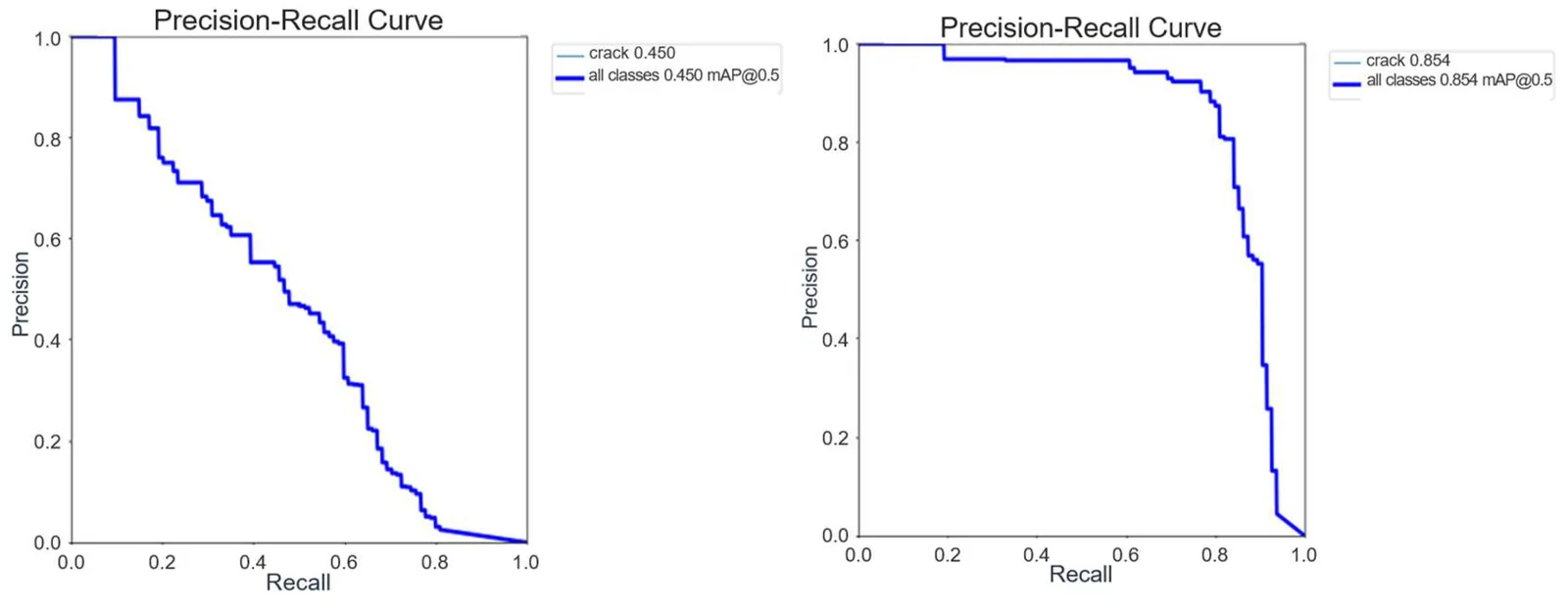
Comparison of segmentation Precision–Recall (PR) curves before and after the improvement of Crack-YOLO.

Figure 12 compares the segmentation PR curves of the baseline YOLOv8-Seg model and the optimized Crack-YOLO model. The segmentation mAP50 increased from 0.450 to 0.854 after the incorporation of the CoT and SM Blocks. The optimized PR curve shifts toward the upper-right region, indicating an improved balance between precision and recall and better segmentation performance for small and irregular fissures.

(4) Visual Prediction Analysis

**Figure 13:**
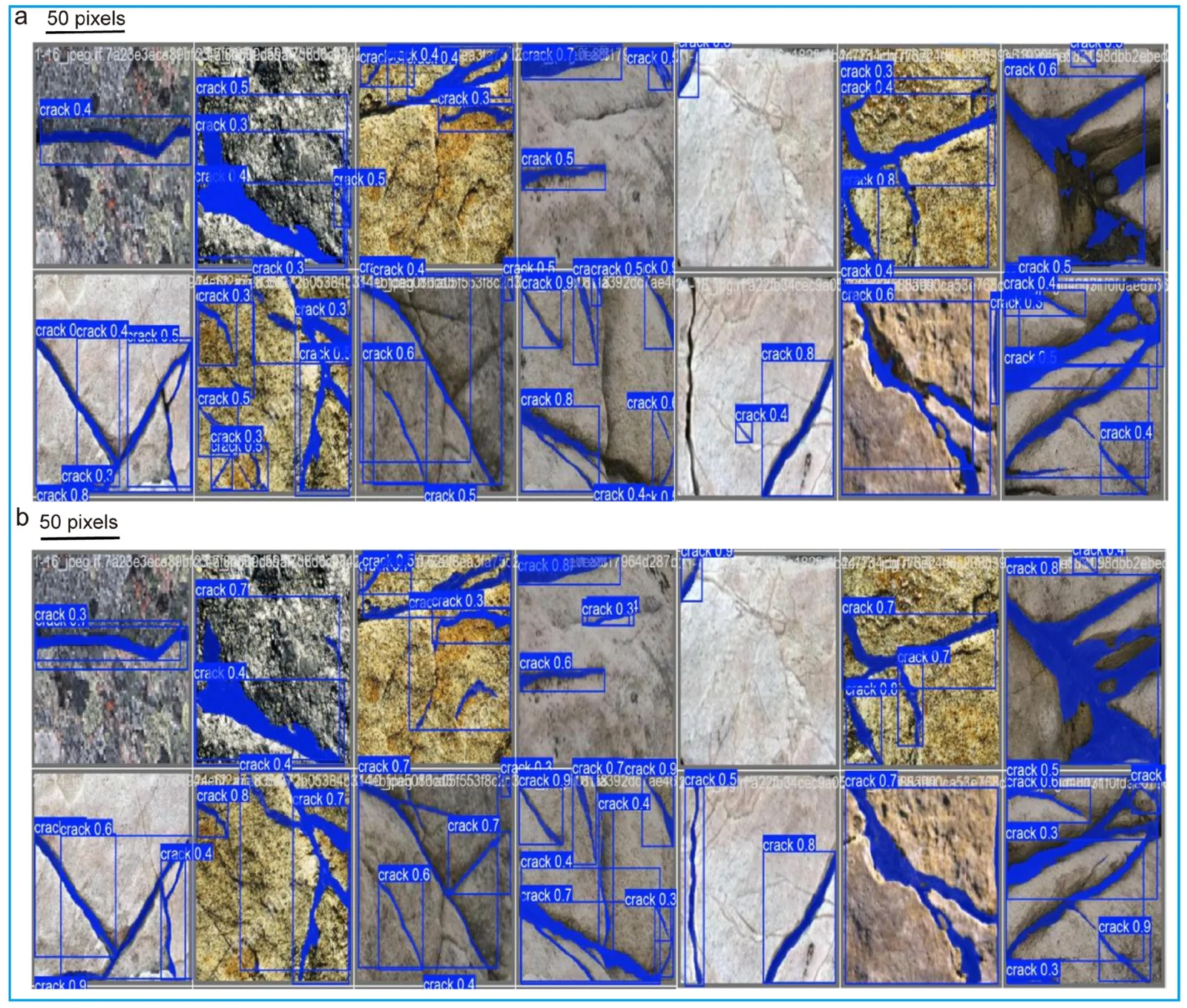
Comparative Visualization of Crack-YOLO Before and After Improvement. (**a**) Instance Segmentation Visualization Before Improvement, (**b**) Instance Segmentation Visualization of Crack-YOLO.

Beyond comparative and ablation experiments, this study incorporates instance segmentation visualization analysis to construct a comprehensive multi-dimensional validation system. In complex-background fissure-segmentation scenarios, visual outcomes can intuitively reflect the model’s feature extraction and boundary localization strengths and limitations. Visual comparison between baseline and optimized models demonstrates evident performance improvements, verifying that the customized modules effectively refine segmentation accuracy and mitigate prediction errors. Such visualization-driven evaluation provides a reliable foundation for network structural optimization and iteration, significantly enhancing the interpretability and credibility of the model’s detection performance. Figure 13 illustrates the instance segmentation results of the Crack-YOLO model before and after optimization.

Structural optimization and embedded dual functional modules contribute to the comprehensive performance enhancement of the Crack-YOLO model. The neighboring region semantic enhancement module strengthens the network’s feature extraction ability and refines the accuracy of crack boundary segmentation. In addition, the texture information enhancement mechanism effectively promotes the model’s perception and recognition of detailed feature information. Benefiting from these improvements, the optimized model achieves superior adaptability for small crack targets and complex background scenarios, which fully verifies the efficacy of the proposed algorithm in feature extraction and semantic comprehension.

### 3.4. Discussion of Comparative Results Between Crack-YOLO and Other YOLO Algorithms

Built upon the YOLOv8-Seg baseline, Crack-YOLO incorporates a contextual semantic enhancement module and a texture information enhancement module. To evaluate its performance, comparative experiments were conducted using selected YOLO-series instance-segmentation models, including YOLOv8-Seg, YOLOv9-Seg, and YOLOv11-Seg.

To ensure a fair comparison, all models were trained and evaluated using the same dataset partition, input image size, training epochs, batch size, optimizer settings, data-augmentation strategy, and evaluation metrics. Table 4 presents a quantitative comparison between Crack-YOLO and selected YOLO-series instance-segmentation models in terms of object detection and instance segmentation performance.

Among the selected baseline models, YOLOv11-Seg achieved the highest segmentation precision of 0.582 but the lowest recall of 0.447, indicating relatively conservative predictions and a greater tendency to miss fissure instances. YOLOv8-Seg showed a comparatively better balance between precision and recall. In contrast, Crack-YOLO improved both precision and recall and achieved the highest mAP values in both instance segmentation and object detection, indicating a more favorable balance between prediction accuracy and detection completeness. This improvement may be attributed to the complementary effects of the CoT Block and SM Block, which enhance contextual semantic representation and weak-texture feature extraction, respectively. Nevertheless, the comparison is limited to the selected YOLO-series instance-segmentation models and the dataset used in this study.

### 3.5. K3M-Based Fissure Skeleton Extraction and Geometric-Parameter Quantification Results

**Figure 14:**
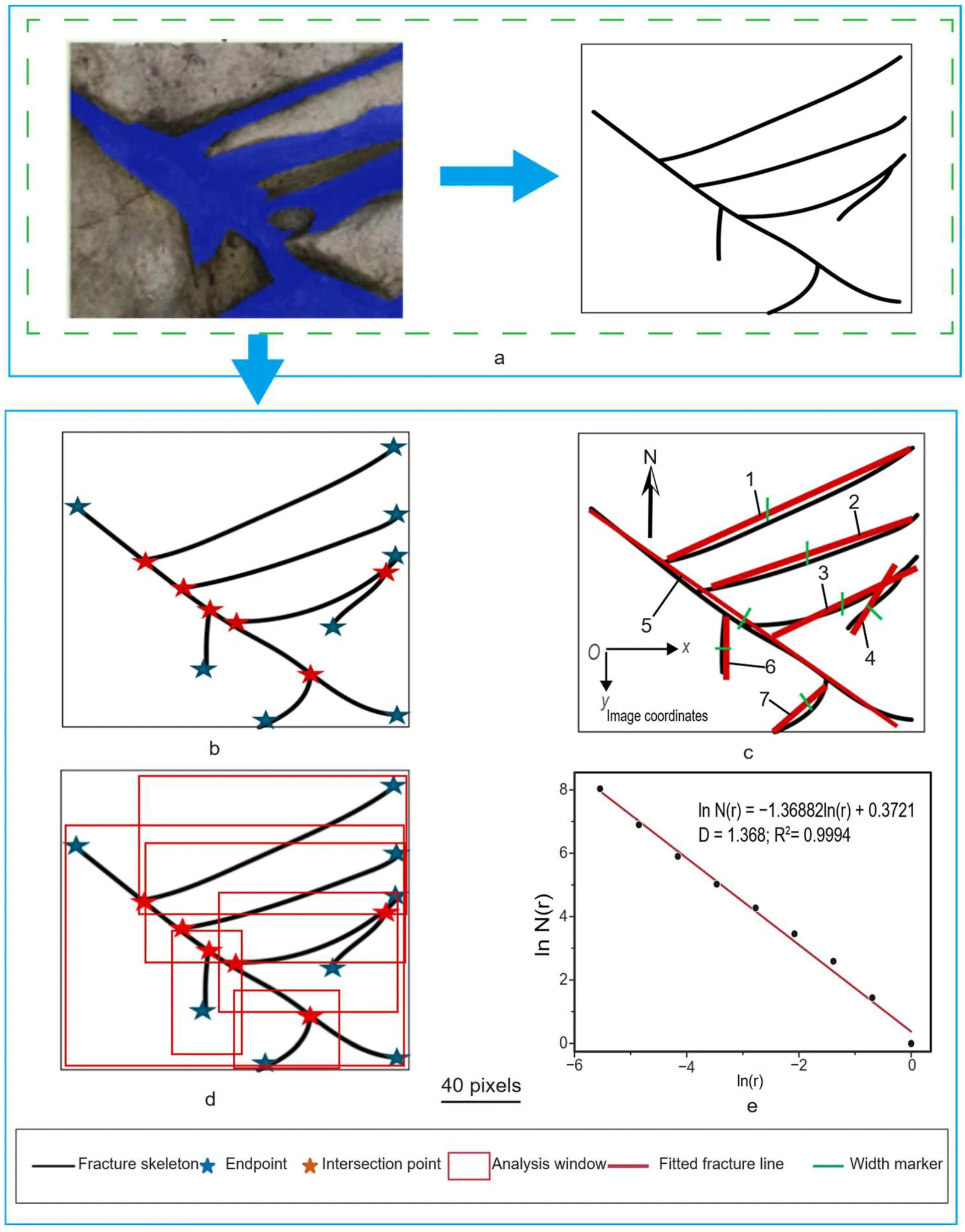
K3M-based fissure skeleton extraction and geometric-parameter quantification: (**a**) fissure segmentation and skeleton extraction; (**b**) endpoint and intersection-point identification; (**c**) fissure numbering and geometric-parameter extraction; (**d**) extraction of individual fissure branches and analysis regions; and (**e**) linear-regression analysis for fractal-dimension calculation. The numbers 1–7 in (**c**) denote the seven representative fissures used for geometric-parameter validation.

A representative image containing seven fissures was selected for the fissure skeleton extraction and geometric-parameter quantification experiment. Figure 14 presents the complete workflow of fissure segmentation, skeleton extraction, and geometric-parameter calculation. First, the pixel-level fissure masks produced by Crack-YOLO were refined using the K3M sequential iteration algorithm to obtain one-pixel-wide fissure skeletons. Subsequently, eight-neighborhood connectivity analysis, skeleton-pixel distance accumulation, linear fitting, mask-area statistics, and the box-counting method were employed to extract the topological, geometric, spatial-distribution, and complexity characteristics of the fissures. The main quantified parameters included the numbers of endpoints and intersection points, fissure length, equivalent average width, azimuth, surface fissure ratio, and fractal dimension. These parameters provide quantitative data support for analyzing fissure development characteristics and evaluating the stability of open-pit mine slopes.

Figure 14a shows the fissure segmentation mask generated by Crack-YOLO and the corresponding skeleton extracted using the K3M algorithm. Figure 14b presents the fissure endpoints and intersection points identified through eight-neighborhood connectivity analysis. Figure 14c shows the numbering of the seven fissures and the extraction results of their lengths, equivalent average widths, and azimuths. According to Table 5, the skeleton lengths of Fissures 1–7 were 66, 52, 22, 20, 135, 18, and 24 pixels, respectively. Their corresponding automatically calculated azimuths were $61.28^{\circ }$, $60.06^{\circ }$, $50.52^{\circ }$, $35.19^{\circ }$, $142.88^{\circ }$, $175.04^{\circ }$, and $45.63^{\circ }$, respectively. Figure 14d illustrates the extraction of individual fissure branches and their corresponding analysis regions, providing the basis for calculating the parameters of individual fissures. Figure 14e presents the box-counting analysis. Nine square-grid sizes were used to cover the fissure skeleton, and linear fitting was performed between lnN(r) and lnr. The resulting fractal dimension of the investigated fissure network was 1.37.

To validate the accuracy of the automated geometric-parameter quantification method, the seven fissures, which differed in length, width, orientation, and morphology, were selected for comparison between the automated results and image-based manual reference measurements, as presented in Table 5. For fissure length, the mean absolute error, root mean square error, and mean absolute percentage error were 0.016 m, 0.018 m, and 2.85%, respectively, with a maximum relative error of 5.88%. For equivalent average width, the corresponding errors were 0.010 m, 0.012 m, and 3.8%, respectively, with a maximum relative error of 7.35%. The mean absolute error and root mean square error of fissure azimuth were $0.90^{\circ }$ and $1.03^{\circ }$, respectively, and the maximum angular error was $1.87^{\circ }$.

The results indicate that, after physical-scale and north-direction calibration of the image, the automated quantification results agreed well with the image-based manual reference measurements. Among the evaluated geometric parameters, the equivalent average width exhibited a relatively larger error because it was more sensitive to local deviations in the segmented fissure boundaries. In comparison, fissure length and azimuth were mainly determined from the skeleton centerlines and were therefore less affected by local boundary deviations. Overall, the integration of Crack-YOLO instance segmentation, K3M-based skeleton extraction, and subsequent image-processing methods can accurately quantify fissure length, equivalent average width, and azimuth.

### 3.6. Engineering Application

**Figure 15:**
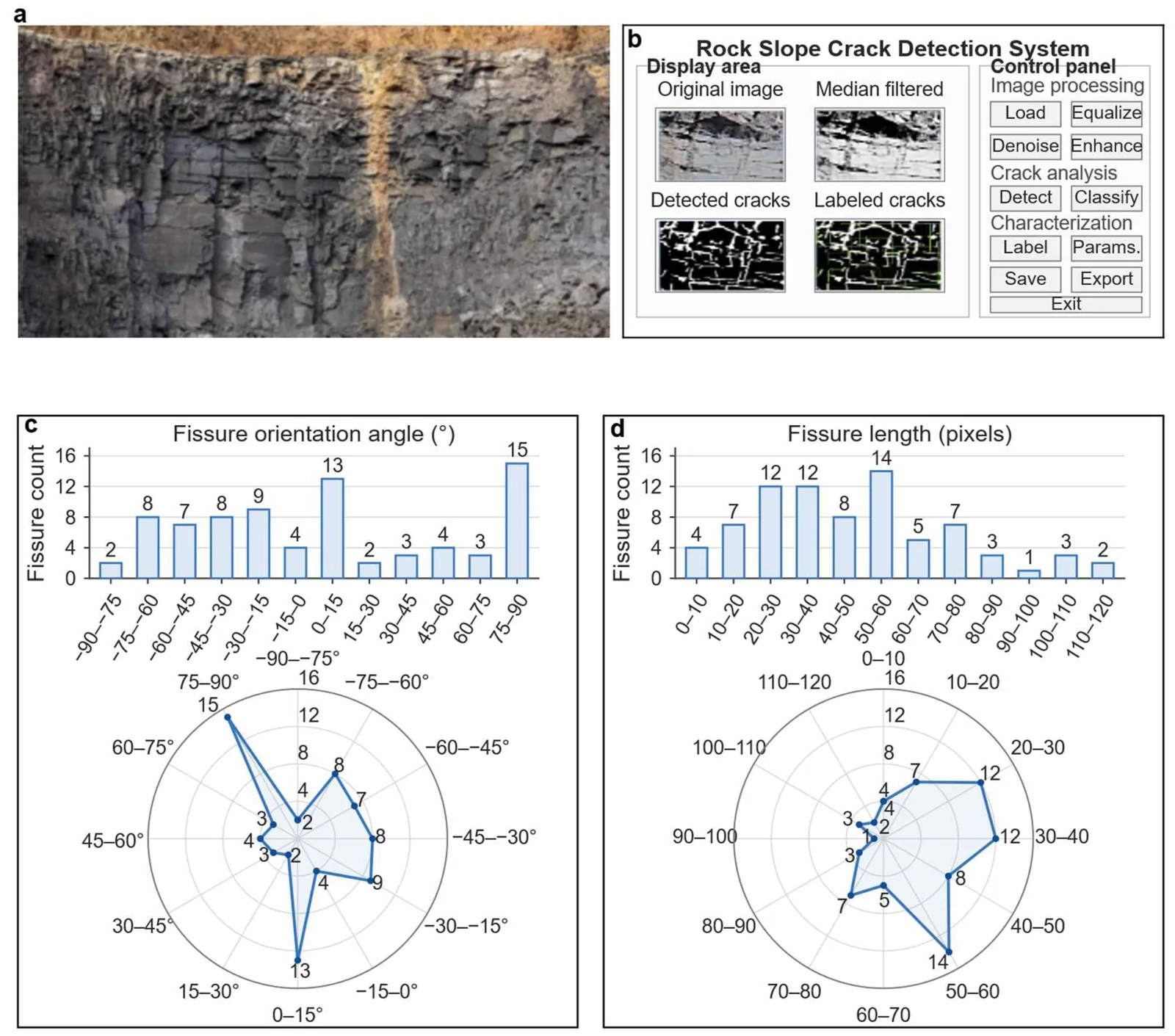
Engineering application results of fissure detection and geometric-parameter quantification for an open-pit rock slope: (**a**) open-pit rock slope; (**b**) system interface and processing results; (**c**) fissure orientation angle distribution; and (**d**) fissure pixel-length distribution.

To integrate fissure detection with geometric-parameter quantification, an intelligent fissure detection and parameter-extraction system was developed for open-pit mine slopes, as shown in Figure 15b. The system integrates Crack-YOLO-based fissure detection and instance segmentation, K3M-based skeleton extraction, geometric-parameter calculation, result visualization, and data export, enabling an integrated workflow from UAV image input to quantitative fissure characterization.

The system was applied to the upper slope of the 1220 bench in Stope 18 of an open-pit mine. The investigated area was approximately 15 m wide and 112 m long, with a slope height of approximately 21 m. The field image was acquired using a DJI Mini 3 UAV (SZ DJI Technology Co., Ltd., Shenzhen, China) at a resolution of 5472 × 3648 pixels and was not included in model training. The system detected 196 fissure instances and required approximately 22 s to complete fissure detection and parameter extraction for the entire image. After completeness screening, 78 fissures with continuous skeletons and valid geometric information were selected for statistical analysis of their azimuths and lengths.

As shown in Figure 15c, the fissure azimuths exhibited a multidirectional distribution. The 0–15° and 75–90° intervals contained relatively large numbers of fissures, with 15 and 13 fissures, respectively, indicating two relatively dominant propagation directions in the investigated area. Figure 15d shows that the fissure pixel lengths were mainly concentrated within the range of 20–60 pixels. A total of 46 fissures fell within this range, accounting for approximately 59.0% of the valid fissures, and the largest number of fissures, 14, occurred in the 50–60-pixel interval. After physical-scale calibration and unit conversion, the actual lengths of the quantified fissures ranged from 0 to 1.8 m. In addition, the surface fissure ratio of the investigated area was 15.51%, and the fractal dimension of the fissure network was 1.25, indicating a certain degree of fissure development and spatial complexity. These results demonstrate that the developed system can achieve automated fissure detection, skeleton extraction, and geometric-parameter quantification in actual open-pit mine slope images, thereby providing quantitative data for characterizing fissure development.

### 3.7. Limitations and Future Work

Although the dataset used in this study contains images collected from multiple open-pit mines and the proposed method was evaluated through a field application, the current validation remains limited to a self-constructed dataset and selected YOLO-series instance-segmentation models. Therefore, the generalization performance of Crack-YOLO on independent external datasets has not yet been fully evaluated. In addition, its robustness under extreme field conditions, such as heavy dust, intense illumination, strong shadows, and complex background interference, requires further investigation. The feasibility of lightweight deployment on edge devices also remains to be examined. Moreover, the instance-level relationship between segmentation quality and errors in fissure geometric parameters has not yet been quantitatively established, and alternative loss functions were not compared under unified experimental conditions.

Future work will focus on expanding the dataset and conducting cross-dataset validation to further assess the generalization performance of the proposed method. Representative CNN- and Transformer-based segmentation models will also be compared under consistent experimental settings. In addition, the relationship between instance-level segmentation metrics and errors in fissure length, equivalent average width, and azimuth will be investigated. Further studies will examine model robustness under complex field conditions, the scale-specific contributions of the CoT and SM Blocks, boundary-aware loss functions, and lightweight deployment for practical field applications.

## 4. Conclusions

This study developed Crack-YOLO, a task-oriented fissure detection and instance-segmentation model based on YOLOv8-Seg, for the intelligent identification and quantitative characterization of rock fissures in open-pit mine slopes. The CoT Block and SM Block were introduced to enhance the contextual semantic representation of complex fissures and the feature extraction of small and weak-texture fissures, respectively. The segmentation results were further integrated with K3M-based skeleton extraction, eight-neighborhood analysis, and physical-scale calibration to quantify fissure geometric parameters. The main conclusions are as follows:

(1) The combined introduction of the CoT and SM Blocks improved the representation of fissures with irregular morphologies, blurred boundaries, and weak-texture features. The ablation results showed that the dual-module configuration outperformed the baseline and single-module configurations, indicating the complementary effects of contextual semantic enhancement and texture-feature enhancement.

(2) Crack-YOLO achieved segmentation precision, recall, mAP50, and mAP50:95 values of 0.896, 0.787, 0.854, and 0.392, respectively. For object detection, the corresponding values were 0.968, 0.862, 0.959, and 0.773. Compared with the original YOLOv8-Seg model, the segmentation mAP50 increased from 0.450 to 0.854, while the detection mAP50 increased from 0.636 to 0.959. Comparisons with the selected YOLO-series instance-segmentation models showed that Crack-YOLO achieved the highest mAP values among the evaluated models on the present dataset. Meanwhile, the segmentation mAP50:95 of 0.392 indicates that further improvement is required for the fine-grained segmentation of small fissures and complex boundaries.

(3) The fissure masks were thinned into one-pixel-wide skeletons using the K3M algorithm. Eight-neighborhood analysis, skeleton-distance calculation, linear fitting, and physical-scale calibration were then employed to quantify fissure length, equivalent average width, and orientation angle. Compared with image-based manual reference measurements, the mean absolute errors of these three parameters were 0.016 m, 0.010 m, and 0.90°, respectively, indicating the feasibility of the proposed geometric-parameter quantification workflow.

(4) In the field engineering application, the system detected 196 fissure instances from a UAV image and completed fissure identification and parameter extraction for the entire image in approximately 22 s. The results indicate that the proposed workflow has potential for fissure identification, geometric-feature quantification, and fissure-development analysis in open-pit mine slopes and may provide quantitative data support for slope-fissure monitoring and stability analysis.

## Figures and Tables

**Table 1:** Dataset Partition and Fissure-Instance Statistics.

Dataset Subset	Number of Images	Proportion (%)	Number of Fissure Instances	Mean Number of Fissure Instances per Image
Training set	3240	90	10,724	3.31
Test set	360	10	1438	3.99
Total	3600	100	12,162	3.38

Note: A fissure instance was defined as a spatially continuous fissure trace in an image. A connected main fissure and its branches were annotated as one instance, whereas spatially disconnected fissure traces were annotated as separate instances. Each image contained at least one annotated fissure instance. The mean number of fissure instances per image was calculated by dividing the total number of fissure instances by the corresponding number of images.

**Table 2:** Confusion Matrix.

Confusion Matrix		Predicted Value	
		Positive	Negative
Truth Value	Positive	True Positive (TP)	False Negative (FN)
	Negative	False Positive (FP)	True Negative (TN)

**Table 3:** Results of the ablation experiments.

CoT Block	SM Block	Seg. Metrics				Det. Metrics			
		P	R	mAP50	mAP 50:95	P	R	mAP50	mAP 50:95
×	×	0.451	0.521	0.450	0.172	0.581	0.596	0.636	0.382
√	×	0.753	0.748	0.74	0.322	0.819	0.819	0.891	0.660
×	√	0.833	0.670	0.780	0.331	0.934	0.753	0.907	0.668
√	√	0.896	0.787	0.854	0.392	0.968	0.862	0.959	0.773

Note: Seg. and Det. denote instance segmentation and object detection, respectively. P and R denote precision and recall, respectively. mAP50 represents the mean average precision at an IoU threshold of 0.50, whereas mAP50:95 represents the mean average precision averaged over IoU thresholds from 0.50 to 0.95.

**Table 4:** Performance comparison with selected YOLO-series instance-segmentation models.

Method	Seg. P	Seg. R	Seg. mAP50	Seg. mAP50:95	Det. P	Det. R	Det. mAP50	Det. mAP50:95
YOLOv8-Seg	0.451	0.521	0.450	0.172	0.581	0.596	0.636	0.382
YOLOv9-Seg	0.467	0.468	0.437	0.157	0.592	0.596	0.614	0.313
YOLOv11-Seg	0.582	0.447	0.442	0.154	0.718	0.532	0.595	0.306
Crack-YOLO	0.896	0.787	0.854	0.392	0.968	0.862	0.959	0.773

Note: Seg. and Det. denote instance segmentation and object detection, respectively. P and R denote precision and recall, respectively. mAP50 represents the mean average precision at an IoU threshold of 0.50, whereas mAP50:95 represents the mean average precision averaged over IoU thresholds from 0.50 to 0.95.

**Table 5:** Comparison and error analysis of automated and manual reference measurements of fissure geometric parameters.

Fissure ID	Automated Length (Pixel)	Automated Length (m)	Manual Fissure Length (m)	Length Error (%)	Automated Equivalent Average Width (Pixel)	Automated Equivalent Average Width (m)	Manual Equivalent Average Width (m)	Width Error (%)	Automated Azimuth (°)	Manual Azimuth (°)	Azimuth Error (°)
1	66	0.99	1.01	1.98	9.03	0.135	0.135	0.00	61.28	63.15	1.87
2	52	0.78	0.75	4	19.81	0.297	0.312	4.81	60.06	59.14	0.92
3	22	0.33	0.33	0	20	0.3	0.317	5.36	50.52	50.86	0.34
4	20	0.30	0.31	3.23	17.4	0.261	0.246	6.10	35.19	34.78	0.41
5	135	2.03	2.01	1	19.54	0.293	0.298	1.68	142.88	143.56	0.68
6	18	0.27	0.26	3.85	13.11	0.197	0.2	1.50	175.04	173.68	1.36
7	24	0.36	0.34	5.88	12.58	0.189	0.204	7.35	45.63	44.93	0.7

Note: Pixel-based measurements were converted into physical dimensions using a calibrated scale of 0.015 m pixel^−1^. Manual image-based measurements were used as reference values for error calculation.

## Data Availability

The data that support the findings of this study are available on request from the corresponding author upon reasonable request.

## References

[1] Ma T.X., Hu X.Q., Liu H.Y., Peng K., Lin Y., Chen Y., Luo K., Xie S., Han C., Chen M. (2025). Elastic modulus prediction for high-temperature treated rock using multi-step hybrid ensemble model combined with coronavirus herd immunity optimizer. Measurement.

[2] Cheng Y., He D.L., Ma T.X., Lin H., Hu X.Q., Liu H.Y. (2024). Hybrid data-driven model and shapley additive explanations for peak dilation angle of rock discontinuities. Mater. Today Commun..

[3] Xie S.J., Jiang Z.Y., Lin H., Ma T.X., Peng K., Liu H.W., Liu B. (2024). A new integrated intelligent computing paradigm for predicting joints shear strength. Geosci. Front..

[4] Xie S.J., Lin H., Ma T.X., Peng K., Sun Z. (2025). Prediction of joint roughness coefficient via hybrid machine learning model combined with principal components analysis. J. Rock Mech. Geotech. Eng..

[5] Alghazo J., Latif G. (2023). AI/ML-based medical image processing and analysis. Diagnostics.

[6] Shehata M., Elhosseini M. (2024). Charting new frontiers: Insights and future directions in ML and DL for image processing. Electronics.

[7] Zhao L., Xu Q.H., Song Z.P., Meng S.Q., Liu S.P. (2024). Dynamic wave tunnel lining GPR images multi-disease detection method based on deep learning. NDT E Int..

[8] Cao H., Ma G., Liu P., Qin X., Wu C., Lu J. (2023). Multi-Factor Analysis on the Stability of High Slopes in Open-Pit Mines. Appl. Sci..

[9] Wu L., He K., Guo L., Sun L. (2022). Analysis of stability law and optimization of slope angle during excavation of deep concave mine slope. PLoS ONE.

[10] Byun H., Kim J., Yoon D., Kang I.-S., Song J.-J. (2021). A deep convolutional neural network for rock fracture image segmentation. Earth Sci. Inform..

[11] Oh H., Garrick N.W., Achenie L.E.K. (1998). Segmentation Algorithm Using Iterative Clipping for Processing Noisy Pavement Images. Imaging Technologies: Techniques and Applications in Civil Engineering, Proceedings of the Second International Conference.

[12] Ouyang A., Dong Q., Wang Y., Liu Y., Li D., Chen Y. (2014). The Classification of Pavement Crack Image Based on Beamlet Algorithm. Computer and Computing Technologies in Agriculture VII.

[13] Qu Z., Ju F.R., Chen S.Q. (2018). Concrete surface crack detection combining structured forest edge detection and percolation model. Comput. Sci..

[14] Zhang Y.L., Xing H.L., Li S.Z., Pang S., Liu J., Zhang R. (2021). Fracture Extraction and Repair of 2D Rock Image Based on Hybrid Algorithm of Ant Colony and Canny Edge Detection Operator. Geotecton. Metallog..

[15] Liu W., Li F.Y., Xiong L.Y., Liu S.L., Wang K. (2016). Shoulder Line Extraction in the Loess Plateau Based on Region Growing Algorithm. J. Geo-Inf. Sci..

[16] Chudasama B., Ovaskainen N., Tamminen J., Nordbäck N., Engström J., Aaltonen I. (2024). Automated mapping of bedrock-fracture traces from UAV-acquired images using U-Net convolutional neural networks. Comput. Geosci..

[17] Chen J., Zhou M., Huang H., Zhang D., Peng Z. (2021). Automated extraction and evaluation of fracture trace maps from rock tunnel face images via deep learning. Int. J. Rock Mech. Min. Sci..

[18] Morales J., Saldivia C., Lobo R., del Pino M., Muñoz M., Caniggia G., Catalán J., Hayde R., Poblete V. (2025). Visual analysis of deep learning semantic segmentation applied to petrographic thin sections. Sci. Rep..

[19] Yaqoob M., Ishaq M., Ansari M.Y., Konagandla V.R.S., Al Tamimi T., Tavani S., Corradetti A., Seers T.D. (2024). GeoCrack: A high-resolution dataset for segmentation of fracture edges in geological outcrops. Sci. Data.

[20] Ruan S., Hu Y., Liu J., Wang J. (2026). An advanced crack detection method for slope management in open-pit mines: Applying enhanced YOLOv8 network. Int. J. Min. Reclam. Environ..

[21] Ruan S., Liu D., Gu Q., Jing Y. (2022). An intelligent detection method for open-pit slope fracture based on the improved Mask R-CNN. J. Min. Sci..

[22] Le Roux R., Sepehri M., Khaksar S., Murray I. (2025). Slope stability monitoring methods and technologies for open-pit mining: A systematic review. Mining.

[23] Zhao H., Jin J., Liu Y., Guo Y., Shen Y. (2024). FSDF: A high-performance fire detection framework. Expert Syst. Appl..

[24] Chen N., Xu Z., Liu Z., Chen Y., Miao Y., Li Q., Hou Y., Wang L. (2022). Data augmentation and intelligent recognition in pavement texture using a deep learning. IEEE Trans. Intell. Transp. Syst..

[25] Wan W., Liao B., Liu Y., Cao J., Zhang B. (2025). Study on an Improved YOLOv8 Algorithm for UAV Image-Based Road Crack Detection. Proceedings of the 2025 IEEE 8th Information Technology and Mechatronics Engineering Conference (ITOEC), Chongqing, China, 14–16 March 2025.

[26] Li M., Cui L. (2024). An improved yolov8-based algorithm for concrete crack detection. Proceedings of the 2024 International Conference on Artificial Intelligence of Things and Computing (AITC 2024), Hangzhou, China, 30 August–1 September 2024.

[27] He X., Liu Y., Wen S., He W. (2025). A Coal Gangue Debris Detection Method Based on YOLOv8-FAPF. Proceedings of the 2025 IEEE 2nd International Conference on Deep Learning and Computer Vision (DLCV), Jinan, China, 6–8 June 2025.

[28] Tian Q., Peng R., Wang F. (2025). Segmentation of Stone Slab Cracks Based on an Improved YOLOv8 Algorithm. Appl. Sci..

[29] Deng Z., Liu Z., Zhao F., Wang M., Zhao H. (2025). SE-YOLOv8: A model for seaside pavement crack detection in remote sensing images. J. Appl. Remote Sens..

[30] Zhang C., Chen X., Liu P., He B., Li W., Song T. (2024). Automated detection and segmentation of tunnel defects and objects using YOLOv8-CM. Tunn. Undergr. Space Technol..

[31] Liu Y., Wang Z.F., Wang Y.Q., Zhou L.-W., Li X.-K., Ding X.-H. (2025). Real-time detection of highway tunnel lining cracks using YOLOv8-DTD with an android application. Autom. Constr..

[32] RangeKing (2023). Brief summary of YOLOv8 model structure. https://github.com/ultralytics/ultralytics/issues/189.

[33] Song R., Sun X., Liu G. (2023). Cross-Modal Generation of Tactile Friction Coefficient From Audio and Visual Measurements by Transformer. IEEE Trans. Instrum. Meas..

[34] Wang T., Zhang H., Jiang D. (2024). CSD-YOLO: A Ship Detection Algorithm Based on a Deformable Large Kernel Attention Mechanism. Mathematics.

